# Bacteriocins of lactic acid bacteria: extending the family

**DOI:** 10.1007/s00253-016-7343-9

**Published:** 2016-02-10

**Authors:** Patricia Alvarez-Sieiro, Manuel Montalbán-López, Dongdong Mu, Oscar P. Kuipers

**Affiliations:** Department of Molecular Genetics, Groningen Biomolecular Sciences and Biotechnology Institute, University of Groningen, Nijenborgh 7, 9747 AG Groningen, The Netherlands; Department of Biochemistry, Groningen Biomolecular Sciences and Biotechnology Institute and Zernike Institute for Advanced Materials, University of Groningen, Nijenborgh 4, 9747 AG Groningen, The Netherlands; School of Biotechnology and Food Engineering, Hefei University of Technology, Hefei, 230009 China

**Keywords:** Bacteriocin, Lactic acid bacteria, Antimicrobial peptides, Lantibiotics, Lasso peptides, Sactipeptides, Circular bacteriocin, Linear azole-containing peptides, Glycocins

## Abstract

Lactic acid bacteria (LAB) constitute a heterogeneous group of microorganisms that produce lactic acid as the major product during the fermentation process. LAB are Gram-positive bacteria with great biotechnological potential in the food industry. They can produce bacteriocins, which are proteinaceous antimicrobial molecules with a diverse genetic origin, posttranslationally modified or not, that can help the producer organism to outcompete other bacterial species. In this review, we focus on the various types of bacteriocins that can be found in LAB and the organization and regulation of the gene clusters responsible for their production and biosynthesis, and consider the food applications of the prototype bacteriocins from LAB. Furthermore, we propose a revised classification of bacteriocins that can accommodate the increasing number of classes reported over the last years.

**Electronic supplementary material:**

The online version of this article (doi:10.1007/s00253-016-7343-9) contains supplementary material, which is available to authorized users.

## Introduction

The production of antagonistic substances by living organisms is a conserved characteristic throughout evolution, constituting an effective ancestral defense mechanism. Bacteriocins are ribosomally produced antimicrobial peptides from bacteria, either processed or not by additional posttranslational modification (PTM) enzymes, and exported to the extracellular medium (Cotter et al. 2005).

Bacteriocins produced by lactic acid bacteria (LAB) are particularly interesting due to the long history of safe use of some of them and the generally regarded as safe (GRAS) and Qualified Presumption of Safety (QPS) status that most LAB possess. LAB are a heterogeneous group of Gram-positive fermentative bacteria belonging to Firmicutes that encompasses various genera (Table 1) (Carr et al. 2002). Although bifidobacteria are not strictly LAB, they have been traditionally studied together and will also be considered in this review.

**Table 1:** Number of putative bacteriocin gene cluster identified in 238 complete genomes

Genera	Class I							Class II	Class III	Total
	Lanthipeptide I	Lanthipeptide II	Cyclic peptide	Sactipeptide	Glycocin	Lasso peptide	LAP			
*Aerococcus* (1)										0
*Bifidobacterium* (31)		2	2							4
*Carnobacterium* (3)		1	6					1		8
*Enterococcus* (12)		3		1	1			13	7	25
*Lactobacillus* (59)			16		23		3	86	76	204
*Lactococcus* (13)	3			7			1	20	1	32
*Leuconostoc* (8)			1					6		7
*Oenococcus* (1)			1							1
*Pediococcus* (3)	1								2	3
*Streptococcus* (105)	16	22	15	7	5	4	33	388	10	500
*Tetragenococcus* (1)									1	1
*Weisella* (1)										0
TOTAL	19	29	41	15	29	4	37	514	97	785

*Numbers in parentheses* () *indicate the number of genomes analyzed per genus*

We aim to provide an overview of the prevalence of bacteriocin classes in LAB. We highlight the classes that have been described in LAB providing examples of the most relevant cases for each class paying attention to the genetics, structure, and mechanism of action. Moreover, we discuss some bacteriocin groups that can be detected in silico in publicly available LAB genomes even though no representative from a LAB has yet been reported. Due to their biotechnological interest, the application of some bacteriocins in food processing is briefly described.

## Classification of bacteriocins from lactic acid bacteria

There are a large number of bacteriocins isolated from nature. Some databases have been created to compile this information (e.g., van Heel et al. 2013). In addition to published bacteriocins, the repertoire of molecules hidden in the genomes that have not been isolated yet represents a valuable source of novel compounds with great potential. Diverse tools have been created that can be used for the automated screening of bacteriocin gene clusters (Blin et al. 2013; van Heel et al. 2013). A total of 238 complete LAB genomes deposited in public databases and belonging to the genera indicated in Table 1 were analyzed using Bagel3. This search resulted in a list of 785 putative bacteriocin gene clusters, including ribosomally produced and posttranslationally modified peptides (RiPPs) that were not previously identified in LAB. In this list, we could observe previously characterized bacteriocins or natural variants, some of them spread among different species, and new putative bacteriocins with no significant homology to known peptides based on the blast results provided (Table S1).

**Fig. 1:**
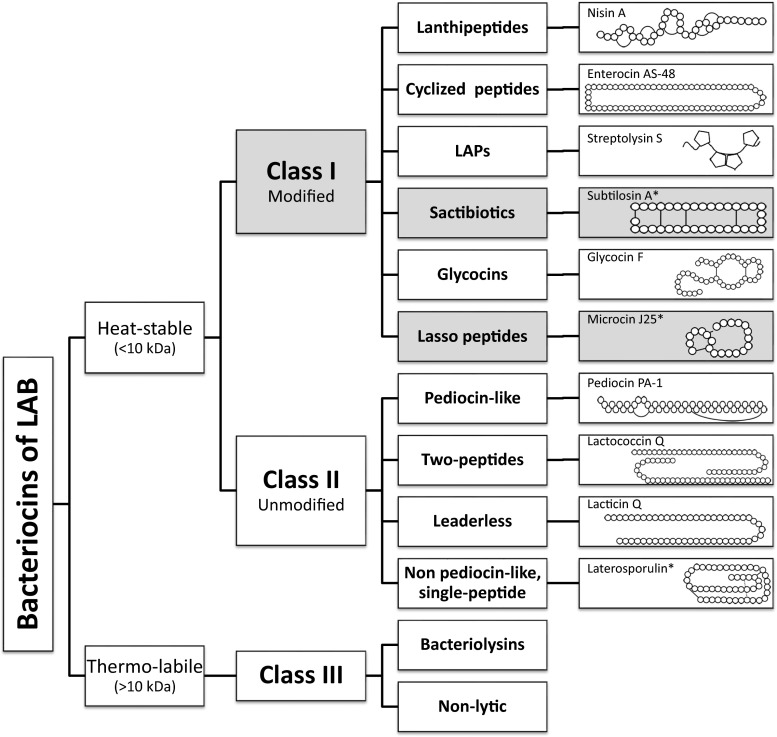
Proposed classification scheme for bacteriocins and their structures. Classes identified in silico are depicted in *gray*. Structure of non-lytic bacteriocins of class III still remains uncharacterized. *Bacteriocins from non-lactic acid bacteria

Since the first classification of LAB bacteriocins, proposed by Klaenhammer (1993), different schemes have been proposed taking it as a basis. New RiPP subgroups with antimicrobial activity produced by bacteria have been discovered that do not fit in either classification in spite of fitting in the definition of bacteriocin. We propose a slightly adjusted classification for LAB that can accommodate the novel subclasses that are appearing, based on the biosynthesis mechanism and biological activity, which is in acoordance with previous proposals (Cotter et al. 2013; Arnison 2013). Although we focus on bacteriocins from LAB, this scheme is also valid for known compounds from other microorganisms (Fig. 1).

### Class I: RiPPs (less than 10 kDa)

This class encompasses all the peptides that undergo enzymatic modification during biosynthesis, which provides molecules with uncommon amino acids and structures that have an impact on their properties (e.g., lanthionine, heterocycles, head-to-tail cyclization, glycosylation). They consist of a leader peptide which serves for enzyme recognition, transport, and keeping the peptide inactive, which is fused to a core peptide (Arnison et al. 2013). The key signatures for an appropriate and systematic definition of novel members of this class have been recently suggested (Medema et al. 2015). Other RiPP subclasses not found in LAB are not further discussed here (for a review see Arnison et al. 2013).

### Class II: unmodified bacteriocins (less than 10 kDa)

This class groups bacteriocins that do not contain unusual modifications. Thus, they do not require enzymes for their maturation other than a leader peptidase and/or a transporter.

### Class III

These are unmodified bacteriocins larger than 10 kDa with bacteriolytic or non-lytic mechanism of action.

## Class I: small posttranslationally modified peptides

### Class Ia. Or lanthipeptides (types I, II, III, and IV)

Lanthipeptides are peptides possessing unusual amino acids, such as lanthionine and/or (methyl)lanthionine (Arnison et al. 2013). Lanthipeptides undergo PTMs, and generally the genes involved in the maturation process are located in the same operon. Based on the PTM enzymes involved in the maturation process, lanthipeptides can be divided into four types, but only types I (LanBC-modified) and II (LanM-modified) can be considered lantibiotics (Knerr and van der Donk 2012). Types III and IV have no known antimicrobial activity and are not further considered here.

**Fig. 2:**
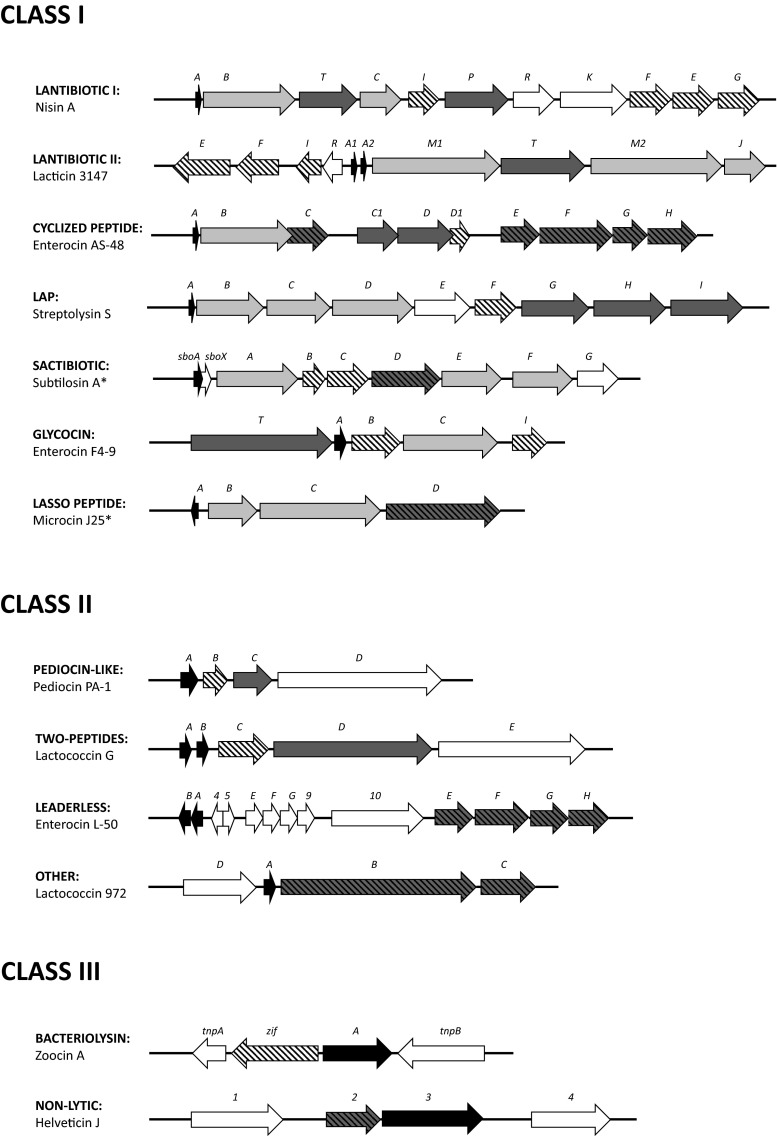
Schematic representation of bacteriocin gene clusters (not drawn to scale). *Black*, structural genes; *dark gray*, transporter genes; *light gray*, modification and maturation genes; *bars*, immunity genes; *white*, others. *Bacteriocins from non-lactic acid bacteria

A great number of different lantibiotics are produced by LAB (Table 1). Among them, nisin, a type I lantibiotic produced by *Lactococcus lactis*, is the best studied. The nisin biosynthetic gene cluster consists of 11 genes (Fig. 2). Promoters transcribing the *nisABTCIP* (biosynthesis and immunity) and *nisFEG* (immunity) operons are controlled by the two-component system NisRK that responds to the nisin concentration in a typical quorum sensing (QS) system (Lubelski et al. 2008). This QS mechanism has been also shown for type II lantibiotics such as bovicin HJ50 (Ni et al. 2011).

Lantibiotic maturation is a process encompassing several enzymatic reactions. The nisin precursor is modified by the dehydratase NisB which dehydrates Ser and Thr via glutamyl-tRNA-dependent glutamylation and elimination (Garg et al. 2013; Ortega et al. 2015). The cyclase NisC promotes the reversible Michael-type nucleophilic attack from the thiol group of a cysteine to an N-terminal-located dehydrated residue rendering the (methyl)lanthionine rings (Lubelski et al. 2008; Yang and van der Donk 2015). Subsequently, the fully modified precursor is transported (NisT) and proteolytically activated (NisP) (Lubelski et al. 2008). For some type I lantibiotics, this step is performed most likely intracellularly (e.g., Pep5) or even by an unknown leader peptidase not associated to the lantibiotic cluster (e.g., subtilin) (Knerr and van der Donk 2012). In type II lantibiotics, the processes of dehydration and cyclization are carried out by a bifunctional LanM enzyme that performs the phosphorylation-elimination reaction on the dehydratable residues and forms the rings (Knerr and van der Donk 2012). Lacticin 3147 from *L. lactis* is one of the best-studied type II lantibiotics. It consists of two peptides, LtnA1 and LtnA2, which are processed by LtnM1 and LtnM2, respectively. Finally, the bifunctional enzyme LtnT removes the leader peptide and translocates the modified peptides (Suda et al. 2012). Recently, the role of the leader peptide of lantibiotics as an activator of the PTM enzymes has been revealed (Oman et al. 2012). Apart from lanthionine rings and dehydrated residues, other modified amino acids have been detected in lantibiotics (Knerr and van der Donk 2012; Ortega et al. 2014; Lohans et al. 2014).

The mechanism of action of most lantibiotics relies on lipid II binding. Nisin inhibits its target removing lipid II from its natural location and subsequent insertion into the cell membrane to form a pore (Breukink et al. 1999; Hasper et al. 2006; Lubelski et al. 2008). Lacticin 3147 also targets lipid II, and a three-step model has been proposed where the α-peptide binds to lipid II, then it is recognized by the β-peptide which inserts into the membrane and forms a pore (Wiedeman et al. 2006; Suda et al. 2012). It has been reported that other type II lantibiotics lack the ability to form pores after interaction with lipid II via a specific binding pocket (Islam et al. 2012), but can strongly induce a cell stress response (Sass et al. 2008). The pore-forming ability of some lantibiotics is compromised by the membrane composition and thickness of the sensitive strain (Wiedeman et al. 2006).

### Class Ib. Or head-to-tail cyclized peptides

Head-to-tail cyclized bacteriocins are a group of RiPPs whose N- and C-termini are linked by a peptide bond, thereby rendering a circular molecule (Fig. 1). All of them contain only alpha helical segments (either 4 or 5) and share a similar structure with a saposin folding (Montalbán-López et al. 2012a; Lohans et al. 2013; Acedo et al. 2015; Himeno et al. 2015).

In spite of their similar structure, two different mechanisms of action are shown for circular bacteriocins, both involving pore formation. In the case of carnocyclin A, it is able to form pores in bacterial membranes in a voltage-dependent manner (Gong et al. 2009). Multimers of carnocyclin A are not found in solution (Martin-Visscher et al. 2009). On the other hand, AS-48 forms dimers in aqueous solution which rearrange in the membrane to bury the hydrophobic core in the lipid bilayer (Cruz et al. 2013; Cebrián et al. 2015). Studies with garvicin ML show that the expression of a maltose-binding protein in *L. lactis* increases the sensitivity to this bacteriocin (Gabrielsen et al. 2012). This constitutes the first report on a putative target for a circular bacteriocin, although its exact role remains to be demonstrated.

The gene cluster of AS-48 is formed by 10 genes including *as-48A* which codes for the structural gene, *as-48B* for a putative cyclase, *as-48C* for a DUF95 protein related to immunity and production (Mu et al. 2014), *as-48C1D* for a putative ABC transporter related to production, *as-48D1* for a typical immunity protein, and *as-48EFGH* for an additional ABC transporter which is immunity related (Fig. 2) (Maqueda et al. 2008). The minimal set for the functional expression of the circular bacteriocin circularin has been determined as *cirABCDE* (equivalent to *as-48ABCDD1*) (Maqueda et al. 2008). The transporter As-48EFGH is not present in some of the known gene clusters indicating that it has a minor role for production and it is related only to immunity (Maqueda et al. 2008; Gabrielsen et al. 2014a). No gene encoding for a putative regulator has been found in the LAB circular bacteriocins described, the only exception being a putative circular bacteriocin cluster detected in silico in *Streptococcus pneumoniae* that contains a putative regulator upstream the structural gene (Maqueda et al. 2008; Bogaardt et al. 2015). The expression of AS-48 requires the expression of a large transcript that encompasses *as-48ABC* which is posttranscriptionally processed, a second transcript including *as-48C1DD1EFGH*, and a third transcript from a weak promoter that transcribes *as-48D1EFGH* (Sánchez-Hidalgo et al. 2011; Cebrián et al. 2014). The typical organization, where the expression of the structural gene is paired to the expression of the immunity and the maturation machinery, is not present in the garvicin ML gene cluster (Gabrielsen et al. 2014b).

The fact that there is not a C-terminal extension in circular bacteriocins as in the case of cyclotides or cyanobactins and that the leader peptide seems to be cleaved off in a separate step could indicate that the cyclization takes place during transport involving the ATPase activity to provide the energy necessary for the peptide bond formation (Montalbán-López et al. 2012a; Craik and Malik 2013; Gabrielsen et al. 2014a; Scholz et al. 2014).

### Class Ic. Or sactibiotics

Sactipeptides (also referred to as sactibiotics when they possess antimicrobial activity) are sulphur-to-α-carbon-containing peptides (Arnison et al. 2013; Mathur et al. 2015). To the best of our knowledge, there has been no sactipeptide from a LAB characterized so far and only putative clusters have been identified in silico (Table 1) (Table S1) (Murphy et al. 2011), awaiting further study.

They show great diversity, with the hairpin structure and the sulfur linkages being the common feature. The best studied, subtilosin A, is a negatively charged circular sactipeptide produced by *Bacillus subtilis* (Fig. 1) (Kawulka et al. 2003; Maqueda et al. 2008). It exhibits 3S-to-α-carbon bonds and displays a broad spectrum activity against diverse bacteria (Montalbán-López et al. 2011; Mathur et al. 2015). Thuricin CD is a narrow-spectrum two-component linear sactibiotic produced by *Bacillus thuringiensis* with potent activity against *Clostridium difficile* (Rea et al. 2010). Thurincin H, also produced by *B. thuringiensis*, is a single peptide with 4S-to-α-carbon bonds (Mathur et al. 2015).

No specific receptor has been identified for sactibiotics. The model, subtilosin A, can partly bury in the membrane of target cells, causing a disorder in the hydrophobic region of the membranes creating transient pores (Noll et al. 2011). On the other hand, thurincin H does not appear to affect the membrane permeability (Wang et al. 2014).

The common features in the sactibiotic gene clusters are the presence of the structural gene(s), immunity proteins, transporters, and S-adenosylmethionine enzymes containing a typical [4S-4Fe] conserved region (Fig. 2) (Flühe et al. 2013).

### Class Id. Or linear azol(in)e-containing peptides

Linear azol(in)e-containing peptides (LAPs) are peptides possessing various combinations of heterocyclic rings of thiazole and (methyl)oxazole, which are derived from cysteine, serine, and threonine residues via ATP-dependent cyclodehydration and subsequent flavin mononucleotide-dependent dehydrogenation (Melby et al. 2011). The most relevant LAB-produced LAP is streptolysin S (Fig. 1) (Cox et al. 2015). Streptolysin S is modified by the cyclodehydratase SagCD. Recently, the SagD-homolog YcaO was shown to be an ATP-dependent enzyme that phosphorylates the amide backbone, although the function of the SacC homolog was not clear (Dunbar et al. 2012). Often, the SagCD analogs in other gene clusters are fused as a single protein. Additionally, the synthesis of streptolysin S requires the dehydrogenase SagB, the protease SagE, the ABC transporter SagGHI, and SagF, probably related with immunity (Lee et al. 2008). The whole set cluster is controlled by a single promoter with a rho-independent terminator behind the structural gene *sagA* (Fig. 2) (Nizet et al. 2000). Additional modifications have been found in other LAP clusters (Lee et al. 2008).

The mechanism of action of LAPs is unclear yet. Microcin B17, from *Escherichia coli*, can inhibit bacterial gyrase under certain conditions in a mechanism similar to quinolones (Heddle et al. 2001).

### Class Ie. Or glycocins

Glycocins are bacteriocins containing glycosylated residue(s) (Arnison et al. 2013). Glycocin F from *Lactobacillus plantarum* was the first glycocin described in LAB (Stepper et al. 2011). Glycocin F is arranged as two alpha helices held together by disulfide bonds (Venugopal et al. 2011). It possesses an N-acetylglucosamine β-O-linked to serine and an N-acetylhexosamine S-linked to the C-terminal cysteine, a very infrequent type of glycosylation (Stepper et al. 2011). Little is known about the mechanism of action of glycocins. The O-linked N-acetylglucosamine could interact reversibly with target cells (Stepper et al. 2011). Apart from glycocin F, enterocin F4-9 from *Enterococcus faecium* has also been described (Fig. 1) (Maky et al. 2015). The biosynthetic gene cluster of enterocin F4-9 consists of five genes (Fig. 2): *enfT*, a putative ABC-transporter; the structural gene *enfA49*; the glycosyltransferase *enfC*; and *enfB* and *enfI*, which resemble a thioldisulfide isomerase and an immunity protein, respectively (Maky et al. 2015). Unlike glycocin F, enterocin F4-9 is assumed to be bacteriostatic (Maky et al. 2015).

### Class If. Or lasso peptides

Lasso peptides are a group of RiPPs that show as a main characteristic the presence of an amide bond between the first amino acid in the core peptide chain and a negatively charged residue in positions +7 to +9 generating a ring that embraces the C-terminal linear part of the polypeptide (Fig. 1) (Arnison et al. 2013; Hegemann et al. 2015). Moreover, lasso peptides display diverse activities which range from antimicrobial to putative antiviral or anticancer (Maksimov et al. 2012). Additional modifications might be naturally present in lasso peptides (Hegemann et al. 2015). Up to date, no lasso peptide from LAB has been reported, but a few are predicted to occur in streptococci (Table 1) (Table S1).

The first antimicrobial lasso peptide characterized was microcin J25, produced by *E. coli* (Fig. 1). A cluster of four genes is required for the production of microcin J25, namely, the structural gene *mcjA*, the immunity determinant *mcjD*, the leader peptidase *mcjB*, and the cyclase *mcjC* (Fig. 2) (Yan et al. 2012). Microcin J25 uses the siderophore transporter FhuA to enter the cell where it acts as a selective transcription inhibitor able to temporarily block the RNA elongation by the RNA polymerase (Mathavan et al. 2014). Additionally, microcin J25 induces the generation of reactive oxygen species that contribute to the inhibition mechanism (Chalon et al. 2009). Similarly, capistruin is a transcription inhibitor (Kuznedelov et al. 2011) whereas lassomycin is a protease inhibitor that targets *Mycobacterium tuberculosis* (Gavrish et al. 2014).

One of the main interests of lasso peptides is their use as peptide scaffolds due to their high stability. Diverse peptide sequences with additional functionalities or even unnatural amino acids can be displayed in lasso peptides (Piscotta et al. 2015; Hegemann et al. 2015).

## Class II: unmodified bacteriocins

### Class IIa. Or pediocin-like bacteriocins

The pediocin-like class IIa bacteriocins are broad spectrum antimicrobials particularly active agains *Listeria* (Kjos et al. 2011). The structure of peptides of class IIa can be divided in two different regions separated by a flexible hinge (Haugen et al. 2008). The cationic N-terminal half contains two cysteine residues joined by a disulfide bridge, and a conserved YGNGVXC motif, which has been suggested to participate in target interaction (Cui et al. 2012). The replacement of this disulfide bridge by hydrophobic interaction can still retain the activity (Sit et al. 2012). The C-terminus is less conserved and seems to be involved in the target cell specificity (Cui et al. 2012).

Class IIa bacteriocins are subdivided into eight groups on the basis of their primary structures (Nissen-Meyer et al. 2009). However, the first and the most extensively studied representative of this class is pediocin PA-1. The gene cluster of pediocin PA-1, like most IIa bacteriocins, is plasmid encoded (Ennahar et al. 1999). The pediocin PA-1 operon contains four genes, namely, the structural gene *pedA*; the immunity determinant *pedB*; and *pedC* and *pedD* which encode an ABC transporter and the accessory protein (Fig. 2). The operon produces two different transcripts; the smaller and most abundant corresponds to the *pedABC* genes, while the second transcript is larger and covers *pedABCD* (Nissen-Meyer et al. 2009; Cui et al. 2012). The leader peptide serves as a recognition signal for the processing and the secretion of the bacteriocin by a dedicated ABC transporter. In a few cases, the bacteriocin is secreted by the sec-dependent translocation system (De Kwaadsteniet et al. 2006). Class IIa bacteriocins can be constitutively produced (e.g., pediocin PA-1) or regulated by a QS system (e.g., sakacin A) (Ennahar et al. 1999).

The mode of action of class IIa bacteriocins comprises three basic steps: pediocin binds to the sugar transporter mannose phosphotransferase system (Man-PTS) receptors, inserts into the cytoplasmic membrane, and finally forms the pore complex (Diep et al. 2007).

### Class IIb. Or two-peptide bacteriocins

Class IIb bacteriocins consist of two very different peptides, and full activity requires the presence of both peptides in about equal quantities (Nissen-Meyer et al. 2010). In some cases, such as lactococcin G from *L. lactis* (Nissen-Meyer et al. 1992), antimicrobial activity requires the presence of both peptides. However, for others such as thermophilin 13 from *Streptococcus thermophilus* (Marciset et al. 1997), individual peptides manifest antimicrobial activity by themselves, although their combination always increases the activity. Exceptionally in enterocin X, this varies in function of the indicator strain (Hu et al. 2010). The peptides can be combined with a complementary peptide from a homologous two-peptide bacteriocin (Oppegård et al. 2007).

Mode-of-action studies of lactococcin G propose that the peptides form a membrane-penetrating helix-helix structure that interacts with a receptor of the membrane of sensitive bacteria (Rogne et al. 2008), causing membrane leakage. Lactococcin G-resistant mutants produced in the lab pointed at the UppP protein, a membrane protein involved in peptidoglycan synthesis, as the putative receptor for lactococcin G and enterocin 1071 (Kjos et al. 2014).

The class IIb bacteriocin production requires at least five different genes, and they might be organized in either one or two different operons. Lactococcin G contains two structural genes codifying the pre-bacteriocins, an immunity gene, a gene that encodes a dedicated ABC transporter, and a gene encoding an accessory protein whose function is still unclear; all of them are arranged in the same operon (Fig. 2) (Oppegård et al. 2010). The structural genes are always produced in equal quantities and found next to each other in the same operon along with only one immunity gene. This fact suggests that the peptides work together as one unit (Nissen-Meyer et al. 2009; Rogne et al. 2008). The production of class IIb bacteriocins is transcriptionally regulated by a QS system of three components, which involves an induction factor, a membrane-associate protein histidine kinase, and response regulators. Plantaricin A, for instance, is the inductor factor of two two-peptide bacteriocins, plantaricin J/K and plantaricin E/F, from *L. plantarum* C11 (Diep et al. 2003).

### Class IIc. Or leaderless bacteriocins

Leaderless bacteriocins are unique as they are synthetized without an N-terminal leader peptide, which usually functions as a recognition sequence for secretion and modification and maintains the bacteriocin inactive inside the producer cell (Liu et al. 2011; Masuda et al. 2012).

One of the best studied and characterized leaderless bacteriocins is the plasmid-encoded two-peptide enterocin L50 from *E. faecium* L50 (Fig. 1) (Cintas et al. 1998). The gene cluster of enterocin L50 encodes 13 open reading frame (ORF) (Fig. 2), including the two structural genes in tandem, some accessory proteins, and four genes highly homologous to the second ABC transporter *as-48EFGH*, which participates in immunity (Franz et al. 2007). The lack of genes encoding immunity proteins is a common feature among leaderless bacteriocins, and the self-immunity mechanism is therefore still unclear (Iwatani et al. 2012).

NMR research has shown that enterocins 7A and 7B are highly homologous to enterocins L50A and L50B and, unlike most linear bacteriocins such as EJ97 (Neira et al. 2010), maintain a defined structure in aqueous conditions (Lohans et al. 2013). Moreover, enterocins 7A and 7B share a structural motif with the circular bacteriocins. Lacticin Q causes membrane leakage without any specific membrane receptor (Yoneyama et al. 2009a). Cationic lacticin Q binds to negatively charged membranes by electrostatic interactions and forms huge toroidal pores that cause leakage (Yoneyama et al. 2009b). In spite of the lack of a docking molecule, the mechanism is selective against several sensitive Gram-positive bacteria due to accumulation of hydroxyl radicals as activity-inducing factor (Li et al. 2013). On the contrary, a zinc-dependent membrane metallopeptidase has been identified as the docking molecule of the leaderless bacteriocin LsbB from *L. lactis* subsp. *lactis* BGMN1-5 (Uzelac et al. 2013). Recently, it has been described for the first time that the expression of LsbB is regulated by a transcription terminator sequence located downstream of the structural gen (Uzelac et al. 2015).

### Class IId. Or non-pediocin-like, single-peptide bacteriocins

Class IId is a heterogeneous group of unrelated single linear peptide bacteriocins with different structures, mechanisms of secretion, and manners of action such as lactococcin 972, lactococcin A, and enterocin B (Franz et al. 2007).

Lactococcin 972 is a heat-sensitive pH-stable peptide active against closely related lactococci species (Martínez et al. 1999). The NMR structure of lactococcin 972 has been recently determined (Turner et al. 2012). The gene cluster is located in a plasmid and comprises the structural gene *lcn972* and two hypothetical genes which could encode a dedicated ABC transporter involved in immunity (Fig. 2) (Campelo et al. 2014). The mature protein is secreted via a sec-dependent system. Two different transcripts are produced: one comprises the whole operon, and the second corresponds to the structural gene (Martínez et al. 1999). The mechanism of action in lactococcin 972 is through the inhibition of the cell wall biosynthesis in lactococci by binding to the cell wall precursor lipid II (Martínez et al. 2000).

Lactococcin A is a narrow spectrum bacteriocin produced by strains of *L. lactis* (Holo et al. 1991). The biosynthesis of lactococcin A involves four different genes: the structural gene (*lcnA*), the immunity gene (*lciA*), and two genes (*lcnC*, *lcnD*) that encode the dedicated ABC transporter system and its accessory protein (Stoddard et al. 1992). The Man-PTS is the target receptor of lactococcin A (Diep et al. 2007).

## Class III

Class III bacteriocins are large-molecular-weight and heat-labile antimicrobial proteins usually composed of different domains. For instance, based on sequence analysis, enterolysin A consists of an N-terminal endopeptidase domain and a C-terminal substrate recognition domain similarly to zoocin A (Nilsen et al. 2003; Lai et al. 2002). Zoocin A, a d-alanyl-l-alanine endopeptidase, is one of the best-characterized LAB bacteriolysins (Fig. 2) (Simmonds et al. 1996). It shows antimicrobial activity against other streptococci by cleaving the peptidoglycan cross-links of the target cell wall (Simmonds et al. 1996). The *zif* gene, close to *zooA*, encodes an immunity protein which adds l-alanine into the peptidoglycan cross-bridges, thus decreasing the ability of zoocin A to degrade the peptidoglycan layer (Gargis et al. 2009).

Millericin B is a murein hydrolase. Its production depends on the expression of three genes encoding millericin B precursor (MilB), immunity protein (MilF), and transporter protein (MilT) (Beukes et al. 2000). Similarly, enterolysin A cleaves within the peptidoglycan of target cells between l-alanine and d-glutamic acid of the stem peptide and between l-lysine of the stem peptide and d-aspartic acid of the interpeptide bridge (Khan et al. 2013).

On the other hand, non-lytic bacteriocins exhibit their bactericidal mode without causing concomitant cell lysis. For instance, dysgalacticin from *S. pyogenes* binds to the glucose- and/or Man-PTS, resulting in the inhibition of the sugar uptake, and also causes a membrane leakage of small molecules (Swe et al. 2009). In contrast, caseicin from *Lactobacillus casei* inhibits the biosynthesis of DNA and proteins of target bacteria (Müller and Radler 1993). Little is known about their genetics. The biosynthesis of helveticin J from *Lactobacillus helveticus* 481 involves at least three ORF, but their specific functions remain still unknown (Fig. 2) (Joerger and Klaenhammer 1990).

## Application in the food industry

Nowadays, consumers ask for safe, healthy, tasting, long shelf-life, and minimally processed food products. LAB are food-grade microorganisms that have been extensively used in fermented foods, and many of them have GRAS and QPS status. As a result, bacteriocins and other metabolites produced by LAB are also generally considered as safe compounds with interesting properties (e.g., stability, antimicrobial activity, lack of toxicity, no flavor alteration) (Carr et al. 2002; Cotter et al. 2005). Until now, only nisin and pediocin PA-1 have been commercialized as food additives. However, other LAB bacteriocins also offer promising perspectives to be used as biopreservatives in food, like for instance the enterocin AS-48 (Sánchez-Hidalgo et al. 2011) or lacticin 3147 (Suda et al. 2012).

Bacteriocins can be added as bacteriocin preparations or by direct inoculation of the bacteriocin-producing strain. The bacteriocin preparation can be a purified or semi-purified bacteriocin added as food preservative, such as nisin which is commonly exploited under the name of Nisaplin™ (Danisco, E234) (Cotter et al. 2005). In fact, nisin is the only bacteriocin licensed as biopreservative in over 50 countries. Bacteriocins can also be added in the form of concentrated fermentate generated from a bacteriocin-producing strain. For instance, ALTA 2431™ (Quest) is a fermentation product from a pediocin PA-1-producing strain (Rodríguez et al. 2002). Bacteriocinogenic strains can be as well directly inoculated into the food as starter, adjunct, or protective cultures. Actually, LAB and, therefore, their bacteriocins, have been empirically applied as starter cultures in the production of traditional foods (Leroy et al. 2006; Alegría et al. 2010). Recently, bacteriocins have also been incorporated into packaging films to control food-borne pathogenic bacteria ensuring a gradual release of bacteriocins into the food and avoiding the inactivation of the bacteriocin by interaction with food components (Guerra et al. 2005). Furthermore, several studies have shown that bacteriocin antimicrobial activity is enhanced against Gram-negative bacteria when combined with physicochemical treatments (hurdle technology) such as high pressure (Pérez Pulido et al. 2015), organic acids (Ukuku and Fett 2004), phenolic compounds (Grande et al. 2007), and pulsed electric fields (Martínez Viedma et al. 2008).

### Enhancement of probiotic action

Many LAB strains are proposed as probiotics, i.e., live microorganisms which, when administered in adequate amounts, confer a health benefit on the host (FAO/WHO 2001). In recent years, several in vitro and in vivo studies have shown that LAB bacteriocins exhibit a protective effect in the gastrointestinal tract (GIT) by excluding pathogens or promoting gut colonization (Corr et al. 2007; Dobson et al. 2012; Kommineni et al. 2015). For instance, the antilisterial Abp118 from *Lactobacillus salivarius* UCC118 protects mice against infection with the pathogen *L. monocytogenes* (Corr et al. 2007), and the *S. mutans* BCS3-L1 strain is able to replace existing *S. mutans* populations and persist over time in the oral cavity, due to the advantage conferred by its bacteriocin, mutacin 1140 (Hillman et al. 2000). Furthermore, Kominneni et al. (2015) proved that niche competition in the GIT is directly influenced by bacteriocin expression by commensal bacteria.

Gastrointestinal infections are a major concern in human health, but antibiotics cause a harmful effect on gut microbiota. Therefore, the anti-infective effect of LAB-producing bacteriocins is a promising alternative to antibiotics, especially for particular cases where other methods are not allowed (e.g., pregnant women) (Hammami et al. 2013).

## Concluding remarks

The concomitant application of bacteriocin-producer LAB or (semi)purified bacteriocins, together with the application of other physicochemical treatments, constitutes an effective method of natural biopreservation in food industry and enables the reduction of other costly or user-unfriendly treatments, while increasing the product self-life.

The discovery of the gene clusters encoding for RiPPs that were previously thought to be non-ribosomally produced but assembled by multimeric enzymatic complexes demonstrates the huge chemical diversity that can be achieved in natural products by the sequential modification of a peptide substrate by specific PTMs. This chemical diversity is related to different properties (i.e., antimicrobial spectrum, stability, potency). The conserved motifs in the PTMs and the core peptides facilitate the high-throughput analysis of (meta)genomic data which can help focus the screening efforts to discover new molecules using diverse alternatives (Montalbán-López et al. 2012b; Hegemann et al. 2015; Rutledge and Challis 2015). In addition to the three bacteriocin classes proposed in the early 1990s (Klaenhammer 1993), our broad genome mining analysis of LAB shows that the repertoire of antimicrobials that are encoded in public sequences could be even broader than expected, with some putative classes not reported so far in LAB (i.e., lasso peptides and sactipeptides), opening up a wide range of possibilities for future applications.

## Electronic supplementary material

ESM 1 (DOCX 104 kb)
