# Supplementary material for: Bacteriocins of lactic acid bacteria: extending the family
Source: Appl Microbiol Biotechnol. 2016 Feb 10;100:2939–51. doi: 10.1007/s00253-016-7343-9 (PMC4786598; doi:10.1007/s00253-016-7343-9)
Supplement: Supplementary file 1 — (DOCX 104 kb) [file 253_2016_7343_MOESM1_ESM.docx]

**Supplementary material**

**Title:** Bacteriocins of lactic acid bacteria: extending the family

**Journal:** Applied Microbiology and Biotechnology

**Authors:** Patricia Alvarez-Sieiro^1,2^, Manuel Montalbán-López^1^, Dongdong Mu^1,3^, Oscar P. Kuipers^1*^

**Affiliation:**

^1^ Department of Molecular Genetics, Groningen Biomolecular Sciences and Biotechnology Institute University of Groningen, Nijenborgh 7, 9747AG, Groningen, The Netherlands.

^2^ Department of Biochemistry, Groningen Biomolecular Sciences and Biotechnology Institute & Zernike Institute for Advanced Materials, University of Groningen, Nijenborgh 4, 9747 AG Groningen, The Netherlands

^3^ School of Biotechnology and Food Engineering, Hefei University of Technology, Hefei, 230009, China.

^*^**Correspondence:** Oscar P. Kuipers, e-mail: [o.p.kuipers@rug.nl](mailto:o.p.kuipers@rug.nl), telephone: 31 50 363 2093, fax number: +31 50 363 2348.

**Supplemental Table S1.** Detailed report of 785 putative bacteriocin genes identified in 238 completed genomes of lactic acid bacteria.

*LAPs, linear azol(in)e-containing peptides; AOI, area of interest; orf, open reading frame.*

| Genus | Species | Subspecies | Strain | AOI;orf | Class | Blast hit |
| --- | --- | --- | --- | --- | --- | --- |
| *Lactococcus* | *L. lactis* | *lactis* | Il1403 | AOI_1;orf020 | Unmodified | Lactococcin_B_(LCN-B)[5e-08] |
| *Lactococcus* | *L. lactis* | *lactis* | Il1403 | AOI_1;orf028 | Unmodified | Lactococcin_B_(LCN-B)[5e-06] |
| *Lactococcus* | *L. lactis* | *lactis* | Il1403 | AOI_2;orf009 | Sactipeptides |  |
| *Lactobacillus* | *L. plantarum* |  | WCFS1 | AOI_1;orf016 | Glycocin | PlnK_(putative)[1e-37] |
| *Lactobacillus* | *L. plantarum* |  | WCFS1 | AOI_1;orf025 | Glycocin | Plantaricin_A[7e-25] |
| *Lactobacillus* | *L. plantarum* |  | WCFS1 | AOI_1;orf033 | Glycocin | Plantaricin_E[7e-37] |
| *Lactobacillus* | *L. plantarum* |  | WCFS1 | AOI_1;smallORF_13 | Glycocin | Plantaricin_N[3e-35] |
| *Lactobacillus* | *L. plantarum* |  | WCFS1 | AOI_1;smallORF_14 | Glycocin | Plantaricin_N[3e-26] |
| *Lactobacillus* | *L. plantarum* |  | WCFS1 | AOI_1;smallORF_17 | Glycocin | Plantaricin_A[3e-29] |
| *Lactobacillus* | *L. plantarum* |  | WCFS1 | AOI_1;smallORF_24 | Glycocin | Plantaricin_F[6e-35] |
| *Lactobacillus* | *L. plantarum* |  | WCFS1 | AOI_1;smallORF_30 | Glycocin | Plantaricin_J[6e-37] |
| *Enterococcus* | *E. faecalis* |  | V583 | AOI_1;orf014 | Lanthipeptide_class_II | Cytolysin_ClyLl[4e-45] |
| *Enterococcus* | *E. faecalis* |  | V583 | AOI_1;orf015 | Lanthipeptide_class_II | Cytolysin_ClyLs[1e-42] |
| *Enterococcus* | *E. faecalis* |  | V583 | AOI_1;orf021 | Unmodified | EJ97 Enterocin[2e-28] |
| *Enterococcus* | *E. faecalis* |  | V583 | AOI_1;orf044 | Glycocin | Enterocin_96[3e-52] |
| *Lactobacillus* | *L. johnsonii* |  | NCC533 | AOI_1;orf022 | Bacteriocin >10kd | Bacteriocin_helveticin_J[2e-57] |
| *Lactobacillus* | *L. johnsonii* |  | NCC533 | AOI_2;orf013 | Unmodified | Lactacin_F,subunit_lafA[8e-52] |
| *Lactobacillus* | *L. johnsonii* |  | NCC533 | AOI_2;orf015 | Unmodified | Lactacin_F,subunit_lafX[9e-42] |
| *Lactobacillus* | *L. johnsonii* |  | NCC533 | AOI_2;orf028 | Unmodified | Penocin_A[3e-10] |
| *Lactobacillus* | *L. johnsonii* |  | NCC533 | AOI_2;orf037 | Unmodified | Pediocin[9e-10] |
| *Lactobacillus* | *L. johnsonii* |  | NCC533 | AOI_3;orf020 | Bacteriocin >10kd | Enterolysin_A[5e-24] |
| *Streptococcus* | *S. thermophilus* |  | LMG18311 | AOI_1;orf022 | Lanthipeptide_class_I |  |
| *Streptococcus* | *S. thermophilus* |  | LMG18311 | AOI_2;orf021 | Unmodified | BlpU[1e-49] |
| *Streptococcus* | *S. thermophilus* |  | LMG18311 | AOI_3;orf021 | Sactipeptides |  |
| *Streptococcus* | *S. thermophilus* |  | CNRZ1066 | AOI_1;orf019 | Lanthipeptide_class_I |  |
| *Streptococcus* | *S. thermophilus* |  | CNRZ1066 | AOI_2;orf014 | Unmodified | BlpK[6e-52] |
| *Streptococcus* | *S. thermophilus* |  | CNRZ1066 | AOI_3;orf019 | Sactipeptides |  |
| *Lactobacillus* | *L. acidophilus* |  | NCFM | AOI_1;orf022 | Bacteriocin >10kd | Enterolysin_A[5e-63] |
| *Lactobacillus* | *L. acidophilus* |  | NCFM | AOI_2;orf013 | Bacteriocin >10kd | Bacteriocin_helveticin_J[0.0] |
| *Lactobacillus* | *L. acidophilus* |  | NCFM | AOI_3;orf025 | Unmodified | Acidocin_J1132_beta_peptide_{N-terminal}[3e-13] |
| *Lactobacillus* | *L. salivarius* |  | UCC118 | AOI_1;orf016 | Bacteriocin >10kd | Enterolysin_A[3e-26] |
| *Lactobacillus* | *L. salivarius* |  | UCC118 | AOI_2;orf016 | Bacteriocin >10kd | Enterolysin_A[4e-25] |
| *Lactobacillus* | *L. salivarius* |  | UCC118 | AOI_3;orf013 | Bacteriocin >10kd | Enterolysin_A[5e-23] |
| *Lactobacillus* | *L. salivarius* |  | UCC118 | AOI_1;orf015 | Unmodified | Salivaricin_P_chain_b[7e-44] |
| *Lactobacillus* | *L. salivarius* |  | UCC118 | AOI_1;orf016 | Unmodified | Salivaricin_P_chain_a[1e-40] |
| *Lactobacillus* | *L. delbrueckii* | *bulgaricus* | ATCC_11842 | AOI_1;orf023 | Bacteriocin >10kd | Enterolysin_A[7e-60] |
| *Pediococcus* | *P. pentosaceous* |  | ATCC_25745 | AOI_1;orf022 | Bacteriocin >10kd | Enterolysin_A[1e-23] |
| *Pediococcus* | *P. pentosaceous* |  | ATCC_25745 | AOI_1;orf032 | Bacteriocin >10kd | Enterolysin_A[3e-21] |
| *Lactobacillus* | *L. casei* |  | ATCC_334 | AOI_1;orf027 | Unmodified | LSEI_2163[2e-26] |
| *Lactobacillus* | *L. casei* |  | ATCC_334 | AOI_2;orf026 | Unmodified | LSEI_2386[8e-30] |
| *Lactobacillus* | *L. casei* |  | ATCC_334 | AOI_2;orf033 | Unmodified | Enterocin_X_chain_beta[9e-11] |
| *Lactobacillus* | *L. casei* |  | ATCC_334 | AOI_3;smallORF_24 | Head_to_tail_cyclized_peptide |  |
| *Lactococcus* | *L. lactis* | *cremoris* | SK11 | AOI_1;orf016 | Unmodified | Garvieacinq_garq[3e-15] |
| *Lactococcus* | *L. lactis* | *cremoris* | SK11 | AOI_1;orf030 | Bacteriocin >10kd | Zoocin_A[1e-19] |
| *Lactococcus* | *L. lactis* | *cremoris* | SK11 | AOI_2;orf019 | Unmodified | Lactococcin_A_(LCN-A)[5e-10] |
| *Oenococcus* | *O. oeni* |  | PSU1 | AOI_1;orf032 | Head_to_tail_cyclized_peptide |  |
| *Lactobacillus* | *L. delbrueckii* | *bulgaricus* | ATCC_BAA365 | AOI_1;orf025 | Bacteriocin >10kd | Enterolysin_A[5e-60] |
| *Lactobacillus* | *L. gasseri* |  | ATCC_33323 | AOI_1;orf018 | Bacteriocin >10kd | Bacteriocin_helveticin_J[1e-61] |
| *Lactobacillus* | *L. gasseri* |  | ATCC_33323 | AOI_2;orf015 | Unmodified | Pediocin[5e-09] |
| *Leuconostoc* | *L. mesenteroides* | *mesenteroides* | ATCC_8293 | AOI_1;orf016 | Unmodified | Enterocin_X_chain_beta[5e-11] |
| *Streptococcus* | *S. thermophilus* |  | LMD_9 | AOI_1;orf020 | Unmodified | Blpk[7e-13] |
| *Streptococcus* | *S. thermophilus* |  | LMD_9 | AOI_1;orf027 | Unmodified | Blpu[1e-49] |
| *Streptococcus* | *S. thermophilus* |  | LMD_9 | AOI_1;orf030 | Unmodified | Blpk[3e-12] |
| *Lactococcus* | *L. lactis* | *cremoris* | MG1363 | AOI_1;orf015 | Unmodified | Garvieacinq_garq[2e-21] |
| *Lactococcus* | *L. lactis* | *cremoris* | MG1363 | AOI_2;orf014 | Sactipeptides |  |
| *Lactobacillus* | *L. reuteri* |  | DSM_20016 | AOI_1;orf020 | Bacteriocin >10kd | Enterolysin_A[3e-26] |
| *Lactobacillus* | *L. helveticus* |  | DPC_4571 | AOI_1;orf013 | Bacteriocin >10kd | Helveticin-J[9e-89] |
| *Lactobacillus* | *L. helveticus* |  | DPC_4571 | AOI_2;orf016 | Bacteriocin >10kd | Helveticin[3e-13] |
| *Lactobacillus* | *L. helveticus* |  | DPC_4571 | AOI_2;orf017 | Bacteriocin >10kd | Helveticin-J[4e-38] |
| *Lactobacillus* | *L. helveticus* |  | DPC_4571 | AOI_3;orf022 | Bacteriocin >10kd | Enterolysin_A[4e-63] |
| *Lactobacillus* | *L. helveticus* |  | DPC_4571 | AOI_4;orf018 | Bacteriocin >10kd | Bacteriocin_helveticin_J[7e-177] |
| *Leuconostoc* | *L. citreum* |  | KM20 | AOI_1;orf016 | Head_to_tail_cyclized_peptide |  |
| *Lactobacillus* | *L. reuteri* |  | JCM_1112 | AOI_1;orf019 | Bacteriocin >10kd | Enterolysin_A[3e-26] |
| *Bifidobacterium* | *B. longum* |  | DJO10A | AOI_1;orf011 | Lanthipeptide_class_II | BLD_1648[9e-44] |
| *Bifidobacterium* | *B. longum* |  | DJO10A | AOI_1;smallORF_10 | Lanthipeptide_class_II | BLD_1648[1e-27] |
| *Lactobacillus* | *L. casei* |  | BL23 | AOI_1;orf019 | Bacteriocin >10kd | Enterolysin_A[9e-22] |
| *Lactobacillus* | *L. casei* |  | BL23 | AOI_2;orf016 | Bacteriocin >10kd | Enterolysin_A[1e-25] |
| *Lactobacillus* | *L. casei* |  | BL23 | AOI_3;orf025 | Unmodified | LSEI_2163[2e-26] |
| *Lactobacillus* | *L. casei* |  | BL23 | AOI_4;orf008 | Unmodified | LSEI_2386[8e-29] |
| *Lactobacillus* | *L. casei* |  | BL23 | AOI_4;orf023 | Unmodified | Enterocin_X_chain_beta[3e-12] |
| *Lactobacillus* | *L. casei* |  | BL23 | AOI_4;orf035 | Unmodified | Enterocin_X_chain_beta[5e-09] |
| *Lactobacillus* | *L. casei* |  | BL23 | AOI_4;orf042 | Unmodified | Carnocin_CP52[1e-20] |
| *Lactobacillus* | *L. casei* |  | BL23 | AOI_4;orf050 | Unmodified | Thermophilin_A[1e-06] |
| *Lactobacillus* | *L. casei* |  | BL23 | AOI_5;orf009 | Head_to_tail_cyclized_peptide |  |
| *Bifidobacterium* | *B. longum* | *infantis* | ATCC_15697 | AOI_5;orf009 | Head_to_tail_cyclized_peptide |  |
| *Lactobacillus* | *L. plantarum* |  | JDM1 | AOI_1;orf019 | Unmodified | Plantaricin_E[4e-28] |
| *Lactobacillus* | *L. rhamnosus* |  | GG | AOI_1;orf020 | Unmodified | Enterocin_X_chain_beta[2e-15] |
| *Lactobacillus* | *L. rhamnosus* |  | GG | AOI_2;orf006 | Unmodified | LSEI_2386[2e-08] |
| *Lactobacillus* | *L. rhamnosus* |  | GG | AOI_2;orf019 | Unmodified | Enterocin_X_chain_beta[6e-13] |
| *Lactobacillus* | *L. rhamnosus* |  | GG | AOI_2;orf039 | Unmodified | Carnocin_CP52[7e-15] |
| *Lactobacillus* | *L. rhamnosus* |  | GG | AOI_3;smallORF_38 | Head_to_tail_cyclized_peptide | |
| *Lactobacillus* | *L. rhamnosus* |  | Lc_705 | AOI_1;orf009 | Unmodified | Carnocin_CP52[6e-06] |
| *Lactobacillus* | *L. rhamnosus* |  | Lc_705 | AOI_1;orf021 | Unmodified | LSEI_2386[2e-08] |
| *Lactobacillus* | *L. rhamnosus* |  | Lc_705 | AOI_1;orf034 | Unmodified | Enterocin_X_chain_beta[8e-13] |
| *Lactobacillus* | *L. rhamnosus* |  | Lc_705 | AOI_2;smallORF_41 | Head_to_tail_cyclized_peptide |  |
| *Lactobacillus* | *L. johnsonii* |  | FI9785 | AOI_1;orf024 | Bacteriocin >10kd | Bacteriocin_helveticin_J[1e-57] |
| *Lactococcus* | *L. lactis* | *lactis* | KF147 | AOI_1;orf030 | Lanthipeptide_class_I |  |
| *Lactococcus* | *L. lactis* | *lactis* | KF147 | AOI_2;orf025 | Unmodified | Lactococcin_A_(LCN-A)[1e-09] |
| *Lactococcus* | *L. lactis* | *lactis* | KF147 | AOI_3;orf010 | Sactipeptides |  |
| *Lactococcus* | *L. lactis* | *lactis* | KF147 | AOI_1;orf023 | Unmodified | Lactococcin_B_(LCN-B)[6e-08] |
| *Lactobacillus* | *L. crispatus* |  | ST1 | AOI_1;orf014 | Bacteriocin >10kd | Helveticin-J[5e-168] |
| *Lactobacillus* | *L. crispatus* |  | ST1 | AOI_2;orf022 | Bacteriocin >10kd | Enterolysin_A[1e-59] |
| *Lactobacillus* | *L. crispatus* |  | ST1 | AOI_3;orf018 | Bacteriocin >10kd | Enterolysin_A[2e-99] |
| *Lactobacillus* | *L. crispatus* |  | ST1 | AOI_4;orf011 | Bacteriocin >10kd | Bacteriocin_helveticin_J[8e-172] |
| *Lactobacillus* | *L. crispatus* |  | ST1 | AOI_5;orf019 | Unmodified | Putative_bacteriocin[3e-08] |
| *Leuconostoc* | *L. gelidum* |  | JB7 | AOI_1;orf017 | Unmodified | Enterocin_X_chain_beta[2e-11] |
| *Leuconostoc* | *L. gelidum* |  | JB7 | AOI_1;orf019 | Unmodified | Enterocin_X_chain_alpha[4e-07] |
| *Leuconostoc* | *L. gelidum* |  | JB7 | AOI_1;orf027 | Unmodified | Putative_bacteriocin[8e-06] |
| *Lactobacillus* | *L. casei* |  | W56 | AOI_1;orf020 | Bacteriocin >10kd | Enterolysin_A[9e-22] |
| *Lactobacillus* | *L. casei* |  | W56 | AOI_2;orf016 | Bacteriocin >10kd | Enterolysin_A[1e-25] |
| *Lactobacillus* | *L. casei* |  | W56 | AOI_3;orf028 | Unmodified | LSEI_2163[2e-26] |
| *Lactobacillus* | *L. casei* |  | W56 | AOI_4;orf008 | Unmodified | LSEI_2386[8e-29] |
| *Lactobacillus* | *L. casei* |  | W56 | AOI_4;orf020 | Unmodified | Enterocin_X_chain_beta[3e-12] |
| *Lactobacillus* | *L. casei* |  | W56 | AOI_4;orf032 | Unmodified | Enterocin_X_chain_beta[5e-09] |
| *Lactobacillus* | *L. casei* |  | W56 | AOI_4;orf041 | Unmodified | Carnocin_CP52[1e-20] |
| *Lactobacillus* | *L. casei* |  | W56 | AOI_4;orf049 | Unmodified | Thermophilin_A[1e-06] |
| *Lactobacillus* | *L. casei* |  | W56 | AOI_5;orf009 | Head_to_tail_cyclized_peptide |  |
| *Carnobacterium* | *C. maltaromaticum* |  | LMA28 | AOI_1;orf028 | Sactipeptides |  |
| *Carnobacterium* | *C. maltaromaticum* |  | LMA28 | AOI_2;orf011 | Head_to_tail_cyclized_peptide |  |
| *Carnobacterium* | *C. maltaromaticum* |  | LMA28 | AOI_2;smallORF_17 | Head_to_tail_cyclized_peptide |  |
| *Carnobacterium* | *C. maltaromaticum* |  | LMA28 | AOI_2;smallORF_18 | Head_to_tail_cyclized_peptide |  |
| *Carnobacterium* | *C. maltaromaticum* |  | LMA28 | AOI_3;orf016 | Unmodified | Carnobacteriocin_BM1_(carnobacteriocinb1)[1e-40] |
| *Lactococcus* | *L. lactis* | *cremoris* | UC5099 | AOI_1;orf018 | Unmodified | Lactococcin_A_(LCN-A)[3e-52] |
| *Lactococcus* | *L. lactis* | *cremoris* | UC5099 | AOI_1;orf021 | Unmodified | Lactococcin_B_(LCN-B)[2e-49] |
| *Lactococcus* | *L. lactis* | *cremoris* | UC5099 | AOI_1;orf026 | Unmodified | Lactococcin_B_(LCN-B)[2e-07] |
| *Lactococcus* | *L. lactis* | *cremoris* | UC5099 | AOI_1;orf051 | Unmodified | Lactococcin_A_(LCN-A)[4e-09] |
| *Lactococcus* | *L. lactis* | *cremoris* | UC5099 | AOI_1;orf021 | Unmodified | Garvieacinq_garq[3e-15] |
| *Lactococcus* | *L. lactis* | *cremoris* | UC5099 | AOI_2;orf021 | Unmodified | Lactococcin_A_(LCN-A)[5e-10] |
| *Enterococcus* | *E. faecium* |  | NRRL_B_2354 | AOI_1;orf016 | Unmodified | Enterocin_X_chain_alpha[3e-40] |
| *Enterococcus* | *E. faecium* |  | NRRL_B_2354 | AOI_1;orf017 | Unmodified | Enterocin_X_chain_beta[1e-36] |
| *Enterococcus* | *E. faecium* |  | NRRL_B_2354 | AOI_1;orf021 | Unmodified | Enterocin_B[2e-49] |
| *Enterococcus* | *E. faecium* |  | NRRL_B_2354 | AOI_1;orf024 | Unmodified | Enterocin_B[1e-06] |
| *Enterococcus* | *E. faecium* |  | NRRL_B_2354 | AOI_2;orf020 | Unmodified | Acidocin_LF221B(gassericink7b)[4e-09] |
| *Enterococcus* | *E. faecium* |  | NRRL_B_2354 | AOI_1;orf011 | Bacteriocin >10kd | Enterolysin_A[6e-29] |
| *Enterococcus* | *E. faecium* |  | NRRL_B_2354 | AOI_2;orf018 | Unmodified | Enterocin_P[2e-35] |
| *Lactobacillus* | *L. plantarum* |  | ZJ316 | AOI_1;orf020 | Unmodified | Plantaricin_NC8-alpha[9e-32] |
| *Lactobacillus* | *L. plantarum* |  | ZJ316 | AOI_1;orf033 | Unmodified | Plantaricin_K[2e-37] |
| *Lactococcus* | *L. lactis* | *lactis* | IO1 | AOI_1;smallORF_8 | Lanthipeptide_class_I | Nisin_Z[6e-38] |
| *Lactococcus* | *L. lactis* | *lactis* | IO1 | AOI_2;orf026 | LAPs |  |
| *Lactococcus* | *L. lactis* | *lactis* | IO1 | AOI_3;orf023 | Unmodified | Lactococcin_A_(LCN-A)[4e-10] |
| *Lactococcus* | *L. lactis* | *lactis* | IO1 | AOI_4;smallORF_1 | Sactipeptides |  |
| *Lactobacillus* | *L. acidophilus* |  | La_14v | AOI_1;orf020 | Bacteriocin >10kd | Enterolysin_A[5e-63] |
| *Lactobacillus* | *L. acidophilus* |  | La_14v | AOI_2;orf013 | Bacteriocin >10kd | Bacteriocin_helveticin_J[0.0] |
| *Lactobacillus* | *L. acidophilus* |  | La_14v | AOI_3;orf020 | Unmodified | Acidocin_J1132_beta_peptide_{N-terminal}[3e-13] |
| *Lactobacillus* | *L. plantarum* | *plantarum* | P8 | AOI_1;orf013 | Unmodified | Plantaricin_E[2e-15] |
| *Lactobacillus* | *L. plantarum* | *plantarum* | P8 | AOI_1;orf014 | Unmodified | Plantaricin_E[4e-07] |
| *Lactobacillus* | *L. reuteri* |  | I5007 | AOI_1;orf017 | Bacteriocin >10kd | Enterolysin_A[1e-25] |
| *Lactobacillus* | *L. casei* |  | LOCK919 | AOI_1;orf033 | Bacteriocin >10kd | Enterolysin_A[5e-25] |
| *Lactobacillus* | *L. casei* |  | LOCK919 | AOI_2;orf026 | Unmodified | Enterocin_X_chain_beta[4e-12] |
| *Lactobacillus* | *L. casei* |  | LOCK919 | AOI_2;orf040 | Unmodified | Enterocin_X_chain_beta[5e-09] |
| *Lactobacillus* | *L. casei* |  | LOCK919 | AOI_2;orf047 | Unmodified | Carnocin_CP52[9e-20] |
| *Lactobacillus* | *L. casei* |  | LOCK919 | AOI_2;orf057 | Unmodified | Thermophilin_A[1e-06] |
| *Lactobacillus* | *L. casei* |  | LOCK919 | AOI_3;smallORF_10 | Head_to_tail_cyclized_peptide |  |
| *Lactobacillus* | *L. johnsonii* |  | N62 | AOI_2;orf014 | Unmodified | Lactacin_F,subunit_lafa[5e-51] |
| *Lactobacillus* | *L. johnsonii* |  | N62 | AOI_2;orf015 | Unmodified | Lactacin_F,subunit_lafx[9e-42] |
| *Lactobacillus* | *L. johnsonii* |  | N62 | AOI_2;orf024 | Unmodified | Penocin_A[3e-10] |
| *Lactobacillus* | *L. johnsonii* |  | N62 | AOI_2;orf031 | Unmodified | Pediocin[2e-11] |
| *Lactobacillus* | *L. johnsonii* |  | N62 | AOI_1;orf021 | Bacteriocin >10kd | Bacteriocin_helveticin_J[1e-57] |
| *Enterococcus* | *E.mundtii* |  | QU25 | AOI_1;orf015 | Unmodified | Mundticin_ATO6[1e-36] |
| *Enterococcus* | *E.mundtii* |  | QU25 | AOI_2,orf017 | Unmodified | Enterolysin_A |
| *Enterococcus* | *E.mundtii* |  | QU25 | AOI_1;orf015 | Unmodified | Enterocin_SE-K4[2e-11] |
| *Enterococcus* | *E.mundtii* |  | QU25 | AOI_1;orf015 | Sactipeptides |  |
| *Lactococcus* | *L.lactis* | *lactis* | KLDS40325 | AOI_2;orf011 | Sactipeptides |  |
| *Lactococcus* | *L.lactis* | *lactis* | KLDS40325 | AOI_1;orf020 | Unmodified | Lactococcin_B_(LCN-B)[7e-09] |
| *Lactococcus* | *L.lactis* | *lactis* | KLDS40325 | AOI_1;orf029 | Unmodified | Lactococcin_B_(LCN-B)[9e-07] |
| *Lactococcus* | *L.lactis* | *cremoris* | KW2 | AOI_2;orf021 | Unmodified | Garvieacinq_garq[4e-21] |
| *Lactobacillus* | *L.paracasei* | *paracasei* | 8700_2 | AOI_3;smallORF_10 | Head_to_tail_cyclized_peptide |  |
| *Lactobacillus* | *L.paracasei* | *paracasei* | 8700_2 | AOI_2;orf028 | Unmodified | Thermophilin_A[6e-10] |
| *Lactobacillus* | *L.paracasei* | *paracasei* | 8700_2 | AOI_1;orf004 | Unmodified | LSEI_2386[8e-30] |
| *Lactobacillus* | *L.paracasei* | *paracasei* | 8700_2 | AOI_1;orf015 | Unmodified | Enterocin_X_chain_beta[1e-11] |
| *Lactobacillus* | *L.paracasei* | *paracasei* | 8700_2 | AOI_1;orf028 | Unmodified | Enterocin_X_chain_beta[2e-09] |
| *Lactobacillus* | *L.paracasei* | *paracasei* | 8700_2 | AOI_1;orf035 | Unmodified | Carnocin_CP52[1e-19] |
| *Lactobacillus* | *L.paracasei* | *paracasei* | 8700_2 | AOI_1;orf042 | Unmodified | Thermophilin_A[1e-06] |
| *Enterococcus* | *E.faecium* |  | Aus0085 | AOI_1;orf013 | Bacteriocin >10kd | Enterolysin_A[2e-28] |
| *Enterococcus* | *E.faecium* |  | Aus0085 | AOI_1;orf019 | Unmodified | Acidocin_LF221B(gassericink7b)[4e-09] |
| *Lactobacillus* | *L.reuteri* |  | TD1 | AOI_1;orf014 | Bacteriocin >10kd | Enterolysin_A[1e-25] |
| *Lactobacillus* | *L.helveticus* |  | CNRZ32 | AOI_3;orf015 | Bacteriocin >10kd | Helveticin-J[2e-65] |
| *Lactobacillus* | *L.helveticus* |  | CNRZ33 | AOI_2;orf014 | Bacteriocin >10kd | Helveticin-J[5e-177] |
| *Lactobacillus* | *L.helveticus* |  | CNRZ34 | AOI_1;orf023 | Bacteriocin >10kd | Enterolysin_A[5e-64] |
| *Lactobacillus* | *L.rhamnosus* |  | LOCK908 | AOI_2;smallORF_41 | Head_to_tail_cyclized_peptide | Ma-2pepb |
| *Lactobacillus* | *L.rhamnosus* |  | LOCK908 | AOI_1;orf005 | Unmodified | LSEI_2386[2e-08] |
| *Lactobacillus* | *L.rhamnosus* |  | LOCK908 | AOI_1;orf019 | Unmodified | Enterocin_X_chain_beta[8e-13] |
| *Lactobacillus* | *L.rhamnosus* |  | LOCK908 | AOI_1;orf040 | Unmodified | Carnocin_CP52[2e-20] |
| *Lactobacillus* | *L.rhamnosus* |  | LOCK900 | AOI_2;smallORF_38 | Head_to_tail_cyclized_peptide |  |
| *Lactobacillus* | *L.rhamnosus* |  | LOCK900 | AOI_1;orf020 | Unmodified | Enterocin_X_chain_beta[3e-13] |
| *Lactobacillus* | *L.rhamnosus* |  | LOCK900 | AOI_1;orf042 | Unmodified | Carnocin_CP52[6e-20] |
| *Leuconostoc* | *L. kimchii* |  | IMSNU_11154 | AOI_1;orf018 | Unmodified | Leucocin_B[3e-14] |
| *Leuconostoc* | *L. gasicomitatum* |  | LMG_18811 | AOI_1;orf024 | Unmodified | Plantaricin_NC8-alpha[5e-11] |
| *Lactobacillus* | *L. casei* |  | Zhang | AOI_1;orf021 | Unmodified | Enterocin_X_chain_beta[4e-12] |
| *Lactobacillus* | *L. casei* |  | Zhang | AOI_1;orf036 | Unmodified | Enterocin_X_chain_beta[5e-09] |
| *Lactobacillus* | *L. casei* |  | Zhang | AOI_1;orf043 | Unmodified | Carnocin_CP52[9e-20] |
| *Lactobacillus* | *L. casei* |  | Zhang | AOI_1;orf055 | Unmodified | Thermophilin_A[1e-06] |
| *Lactobacillus* | *L. casei* |  | Zhang | AOI_2;smallORF_10 | Head_to_tail_cyclized_peptide |  |
| *Lactobacillus* | *L. plantarum* | *plantarum* | ST_III | AOI_1;orf018 | Glyocin | Plantaricin_K[2e-37] |
| *Lactobacillus* | *L. plantarum* | *plantarum* | ST_III | AOI_1;orf034 | Glyocin | Plantaricin_E[4e-28] |
| *Lactobacillus* | *L. plantarum* | *plantarum* | ST_III | AOI_1;smallORF_13 | Glyocin | Plantaricin_N[3e-35] |
| *Lactobacillus* | *L. plantarum* | *plantarum* | ST_III | AOI_1;smallORF_14 | Glyocin | Plantaricin_N[3e-26] |
| *Lactobacillus* | *L. plantarum* | *plantarum* | ST_III | AOI_1;smallORF_17 | Glyocin | Plantaricin_A[3e-29] |
| *Lactobacillus* | *L. plantarum* | *plantarum* | ST_III | AOI_1;smallORF_23 | Glyocin | Plantaricin_E[7e-37] |
| *Lactobacillus* | *L. plantarum* | *plantarum* | ST_III | AOI_1;smallORF_24 | Glyocin | Plantaricin_F[6e-35] |
| *Lactobacillus* | *L. plantarum* | *plantarum* | ST_III | AOI_1;smallORF_30 | Glyocin | Plantaricin_J[6e-37] |
| *Lactobacillus* | *L. amylovorus* |  | GRL_1112 | AOI_1;orf017 | Bacteriocin >10kd | Helveticin-J[2e-57] |
| *Lactobacillus* | *L. amylovorus* |  | GRL_1112 | AOI_1;orf026 | Bacteriocin >10kd | Helveticin-J[8e-92] |
| *Lactobacillus* | *L. amylovorus* |  | GRL_1112 | AOI_2;orf013 | Bacteriocin >10kd | Helveticin-J[2e-55] |
| *Lactobacillus* | *L. amylovorus* |  | GRL_1112 | AOI_3;orf017 | Bacteriocin >10kd | Enterolysin_A[5e-65] |
| *Lactobacillus* | *L. amylovorus* |  | GRL_1112 | AOI_4;orf013 | Bacteriocin >10kd | Bacteriocin_helveticin_J[7e-177] |
| *Lactobacillus* | *L. delbrueckii* | *bulgaricus* | ND02 | AOI_1;orf015 | Bacteriocin >10kd | Helveticin-J[1e-52] |
| *Lactobacillus* | *L. delbrueckii* | *bulgaricus* | ND02 | AOI_2;orf018 | Bacteriocin >10kd | Helveticin-J[4e-101] |
| *Lactobacillus* | *L. acidophilus* |  | 30SC | AOI_1;orf016 | Bacteriocin >10kd | Helveticin-J[2e-92] |
| *Lactobacillus* | *L. acidophilus* |  | 30SC | AOI_2;orf012 | Bacteriocin >10kd | Helveticin-J[5e-47] |
| *Lactobacillus* | *L. acidophilus* |  | 30SC | AOI_3;orf018 | Bacteriocin >10kd | Enterolysin_A[5e-65] |
| *Lactobacillus* | *L. acidophilus* |  | 30SC | AOI_4;orf012 | Bacteriocin >10kd | Bacteriocin_helveticin_J[3e-176] |
| *Lactobacillus* | *L. acidophilus* |  | 30SC | AOI_5;orf018 | Glyocin | Bacteriocin_LS2chaina[1e-09] |
| *Lactobacillus* | *L. acidophilus* |  | 30SC | AOI_5;orf025 | Glyocin | Thermophilin_A[1e-09] |
| *Lactobacillus* | *L. acidophilus* |  | 30SC | AOI_5;orf028 | Glyocin | Thermophilin_A[6e-06] |
| *Lactobacillus* | *L. acidophilus* |  | 30SC | AOI_5;orf030 | Glyocin | Amylovorin[7e-42] |
| *Lactobacillus* | *L. acidophilus* |  | 30SC | AOI_5;orf031 | Glyocin | Lactacin_F,subunit_lafa[1e-19] |
| *Lactobacillus* | *L. acidophilus* |  | 30SC | AOI_5;orf034 | Glyocin |  |
| *Lactobacillus* | *L. acidophilus* |  | 30SC | AOI_5;smallORF_33 | Glyocin | Bacteriocin_LS2chainb[8e-07] |
| *Lactobacillus* | *L. amylovorus* |  | GRL_1112 | AOI_1;orf017 | LAPs |  |
| *Carnobacterium* |  |  | 17_4 | AOI_1;orf022 | Head_to_tail_cyclized_peptide |  |
| *Carnobacterium* |  |  | 17_4 | AOI_1;smallORF_10 | Head_to_tail_cyclized_peptide |  |
| *Carnobacterium* |  |  | 17_4 | AOI_1;smallORF_9 | Head_to_tail_cyclized_peptide |  |
| *Lactobacillus* | *L. buchneri* |  | NRRL_B_30929 | AOI_1;orf027 | Head_to_tail_cyclized_peptide |  |
| *Lactobacillus* | *L. kefiranofaciens* |  | ZW3 | AOI_1;orf018 | Bacteriocin >10kd | Bacteriocin_helveticin_J[9e-169] |
| *Lactobacillus* | *L. kefiranofaciens* |  | ZW3 | AOI_2;orf021 | Bacteriocin >10kd | Enterolysin_A[3e-70] |
| *Lactobacillus* | *L. reuteri* |  | SD2112 | AOI_1;orf014 | Bacteriocin >10kd | Enterolysin_A[9e-26] |
| *Lactobacillus* | *L. reuteri* |  | SD2112 | AOI_2;orf015 | Bacteriocin >10kd | Enterolysin_A[4e-23] |
| *Enterococcus* | *E. hirae* |  | ATCC_9790 | AOI_1;orf016 | Bacteriocin >10kd | Enterolysin_A[1e-30] |
| *Lactobacillus* | *L. ruminis* |  | ATCC_27782 | AOI_1;orf029 | Unmodified | Coagulina[3e-18] |
| *Tetragenococcus* | *T. halophilus* |  | NBRC_12172 | AOI_1;orf025 | Bacteriocin >10kd | Zoocin_A[2e-23] |
| *Pediococcus* | *P. claussenii* |  | ATCC_BAA344 | AOI_1;smallORF_7 | Lanthipeptide_class_II | Salivaricina[3e-07] |
| *Enterococcus* | *E. faecium* |  | Aus0004 | AOI_1;orf019 | Unmodified | Hiracin_JM79[4e-28] |
| *Enterococcus* | *E. faecium* |  | Aus0004 | AOI_1;orf013 | Bacteriocin >10kd | Enterolysin_A[2e-28] |
| *Bifidobacterium* | *B. longum* | *infantis* | ATCC_15697 | AOI_1;smallORF_7 | Head_to_tail_cyclized_peptide |  |
| *Enterococcus* | *E. faecalis* |  | 62 | AOI_1;orf014 | Unmodified | Uvib[4e-09] |
| *Lactobacillus* | *L. helveticus* |  | H10 | AOI_1;orf019 | Bacteriocin >10kd | Helveticin[6e-22] |
| *Lactobacillus* | *L. helveticus* |  | H10 | AOI_1;orf020 | Bacteriocin >10kd | Helveticin-J[5e-15] |
| *Lactobacillus* | *L. helveticus* |  | H10 | AOI_2;orf017 | Bacteriocin >10kd | Helveticin-J[3e-54] |
| *Lactobacillus* | *L. helveticus* |  | H10 | AOI_3;orf020 | Bacteriocin >10kd | Bacteriocin_helveticin_J[5e-177] |
| *Lactobacillus* | *L. helveticus* |  | H10 | AOI_4;orf019 | Bacteriocin >10kd | Helveticin-J[4e-60] |
| *Lactobacillus* | *L. helveticus* |  | H10 | AOI_5;orf021 | Bacteriocin >10kd | Enterolysin_A[2e-25] |
| *Lactobacillus* | *L. helveticus* |  | H10 | AOI_5;orf022 | Bacteriocin >10kd | Enterolysin_A[3e-23] |
| *Lactobacillus* | *L. helveticus* |  | H10 | AOI_6;orf019 | Bacteriocin >10kd | Helveticin-J[6e-59] |
| *Lactobacillus* | *L. delbrueckii* | *bulgaricus* | 2038 | AOI_1;orf021 | Bacteriocin >10kd | Enterolysin_A[2e-61] |
| *Lactobacillus* | *L. amylovorus* |  | GRL1118 | AOI_1;orf019 | Bacteriocin >10kd | Helveticin-J[2e-57] |
| *Lactobacillus* | *L. amylovorus* |  | GRL1118 | AOI_1;orf026 | Bacteriocin >10kd | Helveticin-J[2e-92] |
| *Lactobacillus* | *L. amylovorus* |  | GRL1118 | AOI_2;orf012 | Bacteriocin >10kd | Helveticin-J[3e-55] |
| *Lactobacillus* | *L. amylovorus* |  | GRL1118 | AOI_3;orf015 | Bacteriocin >10kd | Enterolysin_A[5e-65] |
| *Lactobacillus* | *L. amylovorus* |  | GRL1118 | AOI_4;orf011 | Bacteriocin >10kd | Bacteriocin_helveticin_J[6e-177] |
| *Lactobacillus* | *L. casei* |  | LC2W | AOI_1;orf018 | Bacteriocin >10kd | Enterolysin_A[9e-22] |
| *Lactobacillus* | *L. casei* |  | LC2W | AOI_2;orf014 | Bacteriocin >10kd | Enterolysin_A[1e-25] |
| *Lactobacillus* | *L. casei* |  | LC2W | AOI_3;orf007 | Unmodified | LSEI_2386[8e-29] |
| *Lactobacillus* | *L. casei* |  | LC2W | AOI_3;orf018 | Unmodified | Enterocin_X_chain_beta[3e-12] |
| *Lactobacillus* | *L. casei* |  | LC2W | AOI_3;orf032 | Unmodified | Enterocin_X_chain_beta[5e-09] |
| *Lactobacillus* | *L. casei* |  | LC2W | AOI_3;orf040 | Unmodified | Carnocin_CP52[1e-20] |
| *Lactobacillus* | *L. casei* |  | LC2W | AOI_3;orf049 | Unmodified | Thermophilin_A[1e-06] |
| *Lactobacillus* | *L. casei* |  | LC2W | AOI_4;orf009 | Head_to_tail_cyclized_peptide |  |
| *Lactobacillus* | *L. casei* |  | BD_II | AOI_1;orf018 | Bacteriocin >10kd | Enterolysin_A[9e-22] |
| *Lactobacillus* | *L. casei* |  | BD_II | AOI_2;orf015 | Bacteriocin >10kd | Enterolysin_A[1e-25] |
| *Lactobacillus* | *L. casei* |  | BD_II | AOI_3;orf024 | Unmodified | LSEI_2163[2e-26] |
| *Lactobacillus* | *L. casei* |  | BD_II | AOI_4;orf015 | Unmodified | LSEI_2386[8e-29] |
| *Lactobacillus* | *L. casei* |  | BD_II | AOI_4;orf027 | Unmodified | Enterocin_X_chain_beta[3e-12] |
| *Lactobacillus* | *L. casei* |  | BD_II | AOI_4;orf039 | Unmodified | Enterocin_X_chain_beta[5e-09] |
| *Lactobacillus* | *L. casei* |  | BD_II | AOI_4;orf046 | Unmodified | Carnocin_CP52[1e-20] |
| *Lactobacillus* | *L. casei* |  | BD_II | AOI_4;orf054 | Unmodified | Thermophilin_A[1e-06] |
| *Lactobacillus* | *L. casei* |  | BD_II | AOI_5;orf009 | Head_to_tail_cyclized_peptide |  |
| *Lactobacillus* | *L. johnsonii* |  | DPC_6026 | AOI_1;orf021 | Bacteriocin >10kd | Bacteriocin_helveticin_J[4e-56] |
| *Lactobacillus* | *L. johnsonii* |  | DPC_6026 | AOI_2;orf013 | Unmodified | Lactacin_F,subunit_lafa[5e-51] |
| *Lactobacillus* | *L. johnsonii* |  | DPC_6026 | AOI_2;orf015 | Unmodified | Lactacin_F,subunit_lafx[9e-42] |
| *Lactobacillus* | *L. johnsonii* |  | DPC_6026 | AOI_2;orf028 | Unmodified | Bacteriocin_31[3e-10] |
| *Lactobacillus* | *L. johnsonii* |  | DPC_6026 | AOI_2;orf037 | Unmodified | Pediocin[9e-10] |
| *Lactobacillus* | *L. salivarius* |  | CECT_5713 | AOI_1;orf014 | Bacteriocin >10kd | Enterolysin_A[3e-25] |
| *Lactobacillus* | *L. salivarius* |  | CECT_5713 | AOI_2;orf015 | Bacteriocin >10kd | Enterolysin_A[4e-25] |
| *Lactobacillus* | *L. salivarius* |  | CECT_5713 | AOI_3;orf011 | Bacteriocin >10kd | Enterolysin_A[3e-23] |
| *Lactobacillus* | *L. rhamnosus* |  | GG | AOI_1;orf020 | Unmodified | Enterocin_X_chain_beta[2e-15] |
| *Lactobacillus* | *L. rhamnosus* |  | GG | AOI_2;orf006 | Unmodified | LSEI_2386[2e-08] |
| *Lactobacillus* | *L. rhamnosus* |  | GG | AOI_2;orf020 | Unmodified | Enterocin_X_chain_beta[6e-13] |
| *Lactobacillus* | *L. rhamnosus* |  | GG | AOI_2;orf040 | Unmodified | Carnocin_CP52[7e-15] |
| *Lactobacillus* | *L. rhamnosus* |  | GG | AOI_3;smallORF_38 | Head_to_tail_cyclized_peptide |  |
| *Lactococcus* | *L. lactis* | *lactis* | CV56 | AOI_1;smallORF_13 | Lanthipeptide_class_I | Nisin_A[4e-38] |
| *Lactococcus* | *L. lactis* | *lactis* | CV56 | AOI_2;orf009 | Sactipeptides |  |
| *Lactobacillus* | *L. rhamnosus* |  | ATCC_8530 | AOI_1;orf009 | Unmodified | Carnocin_CP52[6e-06] |
| *Lactobacillus* | *L. rhamnosus* |  | ATCC_8530 | AOI_1;orf020 | Unmodified | LSEI_2386[2e-08] |
| *Lactobacillus* | *L. rhamnosus* |  | ATCC_8530 | AOI_1;orf034 | Unmodified | Enterocin_X_chain_beta[8e-13] |
| *Lactobacillus* | *L. rhamnosus* |  | ATCC_8530 | AOI_2;orf031 | Unmodified | Pediocin[1e-38] |
| *Lactobacillus* | *L. rhamnosus* |  | ATCC_8530 | AOI_3;smallORF_40 | Head_to_tail_cyclized_peptide |  |
| *Lactococcus* | *L. lactis* | *cremoris* | A76 | AOI_1;orf014 | Unmodified | Lactococcin_A_(LCN-A)[5e-10] |
| *Lactococcus* | *L. lactis* | *cremoris* | A76 | AOI_2;orf016 | Unmodified | Garvieacinq_garq[3e-15] |
| *Lactobacillus* | *L. salivarius* |  | CECT_5713 | AOI_1;orf015 | Unmodified | Salivaricin_P_chain_b[7e-44] |
| *Lactobacillus* | *L. salivarius* |  | CECT_5713 | AOI_1;orf016 | Unmodified | Salivaricin_P_chain_a[1e-40] |
| *Streptococcus* | *S. thermophilus* |  | ND03 | AOI_1;smallORF_6 | Lanthipeptide_class_I |  |
| *Streptococcus* | *S. thermophilus* |  | ND03 | AOI_2;orf015 | Unmodified | Blpk[1e-39] |
| *Streptococcus* | *S. thermophilus* |  | ND03 | AOI_2;orf023 | Unmodified | Blpu[2e-48] |
| *Streptococcus* | *S. thermophilus* |  | ND03 | AOI_2;orf026 | Unmodified | Blpk[3e-12] |
| *Streptococcus* | *S. thermophilus* |  | JIM_8232 | AOI_1;orf018 | Unmodified | Blpk[7e-13] |
| *Streptococcus* | *S. thermophilus* |  | JIM_8232 | AOI_1;orf024 | Unmodified | Blpu[2e-48] |
| *Streptococcus* | *S. thermophilus* |  | JIM_8232 | AOI_1;orf026 | Unmodified | Blpk[3e-12] |
| *Streptococcus* | *S. thermophilus* |  | JIM_8232 | AOI_2;orf017 | Sactipeptides |  |
| *Streptococcus* | *S. thermophilus* |  | MN_ZLW_002 | AOI_1;smallORF_8 | Lanthipeptide_class_I |  |
| *Streptococcus* | *S. thermophilus* |  | MN_ZLW_002 | AOI_2;orf020 | Sactipeptides |  |
| *Streptococcus* | *S. thermophilus* |  | MN_ZLW_002 | AOI_3;orf017 | Unmodified | Blpk[1e-39] |
| *Streptococcus* | *S. thermophilus* |  | MN_ZLW_002 | AOI_3;orf024 | Unmodified | Blpu[2e-48] |
| *Streptococcus* | *S. thermophilus* |  | MN_ZLW_002 | AOI_3;orf027 | Unmodified | Blpk[3e-12] |
| *Lactococcus* | *L. lactis* | *cremoris* | NZ9000 | AOI_1;orf015 | Unmodified | Garvieacinq_garq[2e-21] |
| *Lactococcus* | *L. lactis* | *cremoris* | NZ9000 | AOI_2;orf014 | Sactipeptides |  |
| *Enterococcus* | *E. faecium* |  | DO | AOI_1;orf018 | Bacteriocin >10kd | Enterolysin_A[1e-28] |
| *Enterococcus* | *E. hirae* |  | ATCC_9790 | AOI_1;orf015 | Lanthipeptide_class_II |  |
| *Enterococcus* | *E. hirae* |  | ATCC_9790 | AOI_2;orf028 | Bacteriocin >10kd | Enterolysin_A[7e-28] |
| *Enterococcus* | *E. faecalis* |  | D32 | AOI_1;orf016 | Bacteriocin >10kd | Enterolysin_A[0.0] |
| *Lactobacillus* | *L. helveticus* |  | R0052 | AOI_1;orf014 | Bacteriocin >10kd | Helveticin-J[1e-62] |
| *Lactobacillus* | *L. helveticus* |  | R0052 | AOI_2;orf021 | Bacteriocin >10kd | Bacteriocin_helveticin_J[3e-176] |
| *Lactobacillus* | *L. helveticus* |  | R0052 | AOI_3;orf019 | LAPs |  |
| *Lactobacillus* | *L. helveticus* |  | R0052 | AOI_4;orf029 | Bacteriocin >10kd | Enterolysin_A[2e-91] |
| *Lactobacillus* | *L. helveticus* |  | R0052 | AOI_5;smallORF_23 | LAPs |  |
| *Lactobacillus* | *L. helveticus* |  | R0052 | AOI_6;orf017 | Bacteriocin >10kd | Helveticin-J[2e-57] |
| *Lactobacillus* | *L. buchneri* |  | CD034 | AOI_1;orf027 | Head_to_tail_cyclized_peptide |  |
| *Streptococcus* | *S. pyogenes* |  | SF370 | AOI_1;orf009 | Unmodified | Bacteriocin_like_peptide_associated[5e-52] |
| *Streptococcus* | *S. pyogenes* |  | SF370 | AOI_1;orf010 | Unmodified | Blpi[4e-20] |
| *Streptococcus* | *S. pyogenes* |  | SF370 | AOI_1;orf017 | Unmodified | Putative_bacteriocin[4e-27] |
| *Streptococcus* | *S. pyogenes* |  | SF370 | AOI_1;orf018 | Unmodified | Bacteriocin_likepeptide_associated[4e-41] |
| *Streptococcus* | *S. pyogenes* |  | SF370 | AOI_1;orf022 | Unmodified | Mutacin_IV[4e-11] |
| *Streptococcus* | *S. pyogenes* |  | SF370 | AOI_2;smallORF_4 | LAPs |  |
| *Streptococcus* | *S. pneumoniae* |  | TIGR4 | AOI_1;orf010 | Unmodified | Blpu[7e-47] |
| *Streptococcus* | *S. pneumoniae* |  | TIGR4 | AOI_2;orf003 | Unmodified | Blpi[8e-42] |
| *Streptococcus* | *S. pneumoniae* |  | TIGR4 | AOI_2;orf005 | Unmodified | Blpj[2e-49] |
| *Streptococcus* | *S. pneumoniae* |  | TIGR4 | AOI_2;orf006 | Unmodified | Blpk[6e-50] |
| *Streptococcus* | *S. pneumoniae* |  | TIGR4 | AOI_2;orf014 | Unmodified | Blpm[2e-57] |
| *Streptococcus* | *S. pneumoniae* |  | TIGR4 | AOI_2;orf015 | Unmodified | Blpn[7e-34] |
| *Streptococcus* | *S. pneumoniae* |  | TIGR4 | AOI_2;orf016 | Unmodified | Blpo[2e-32] |
| *Streptococcus* | *S. pneumoniae* |  | TIGR4 | AOI_3;orf010 | Lanthipeptide_class_II |  |
| *Streptococcus* | *S. pneumoniae* |  | R6 | AOI_1;orf012 | Unmodified | Blpu[4e-34] |
| *Streptococcus* | *S. pneumoniae* |  | R6 | AOI_2;orf011 | Unmodified | Lactococcin_972_(Lcn972)[1e-08] |
| *Streptococcus* | *S. pneumoniae* |  | R6 | AOI_3;orf009 | Lanthipeptide_class_II |  |
| *Streptococcus* | *S. pyogenes* |  | MGAS8232 | AOI_1;orf014 | Unmodified | Blpu[2e-17] |
| *Streptococcus* | *S. pyogenes* |  | MGAS8232 | AOI_1;orf020 | Unmodified | Blpo[7e-07] |
| *Streptococcus* | *S. pyogenes* |  | MGAS8232 | AOI_2;orf009 | Unmodified | Putative_bacteriocin[2e-41] |
| *Streptococcus* | *S. pyogenes* |  | MGAS8232 | AOI_2;orf010 | Unmodified | Bacteriocin_likepeptide_associated[4e-43] |
| *Streptococcus* | *S. pyogenes* |  | MGAS8232 | AOI_2;orf015 | Unmodified | Mutacin_IV[2e-06] |
| *Streptococcus* | *S. pyogenes* |  | MGAS8232 | AOI_3;orf008 | LAPs |  |
| *Streptococcus* | *S. pyogenes* |  | MGAS315 | AOI_1;orf017 | Unmodified | Putative_bacteriocin[8e-51] |
| *Streptococcus* | *S. pyogenes* |  | MGAS315 | AOI_1;orf018 | Unmodified | Bacteriocin_likepeptide_associated[1e-41] |
| *Streptococcus* | *S. pyogenes* |  | MGAS315 | AOI_1;orf022 | Unmodified | Mutacin_IV[4e-06] |
| *Streptococcus* | *S. pyogenes* |  | MGAS315 | AOI_2;smallORF_2 | LAPs |  |
| *Streptococcus* | *S. agalactiae* |  | 2603V | AOI_1;orf007 | Bacteriocin >10kd | Zoocin_A[3e-117] |
| *Streptococcus* | *S. mutans* |  | UA159 | AOI_1;orf011 | Unmodified | Bovicin_255_peptide[1e-08] |
| *Streptococcus* | *S. mutans* |  | UA159 | AOI_1;orf012 | Unmodified | Mutacin_IV[1e-49] |
| *Streptococcus* | *S. mutans* |  | UA159 | AOI_2;orf010 | Unmodified | Putative_bacteriocin[6e-51] |
| *Streptococcus* | *S. mutans* |  | UA159 | AOI_3;orf012 | Unmodified | Thermophilin_A[2e-12] |
| *Streptococcus* | *S. mutans* |  | UA159 | AOI_3;orf018 | Unmodified | Blpu[8e-08] |
| *Streptococcus* | *S. mutans* |  | UA159 | AOI_3;orf020 | Unmodified | Blpk[2e-08] |
| *Streptococcus* | *S. agalactiae* |  | NEM316 | AOI_1;orf009 | Bacteriocin >10kd | Zoocin_A[3e-117] |
| *Streptococcus* | *S. pyogenes* |  | SSI_1 | AOI_2;orf016 | Unmodified | Mutacin_IV[4e-06] |
| *Streptococcus* | *S. pyogenes* |  | SSI_1 | AOI_2;orf020 | Unmodified | Bacteriocin_likepeptide_associated[1e-41] |
| *Streptococcus* | *S. pyogenes* |  | SSI_1 | AOI_2;orf021 | Unmodified | Putative_bacteriocin[8e-51] |
| *Streptococcus* | *S. pyogenes* |  | MGAS10394 | AOI_1;orf012 | Unmodified | Bacteriocin_like_peptide_associated[5e-52] |
| *Streptococcus* | *S. pyogenes* |  | MGAS10394 | AOI_1;orf013 | Unmodified | Blpi[4e-20] |
| *Streptococcus* | *S. pyogenes* |  | MGAS10394 | AOI_1;orf017 | Unmodified | Putative_bacteriocin[8e-51] |
| *Streptococcus* | *S. pyogenes* |  | MGAS10394 | AOI_1;orf018 | Unmodified | Bacteriocin_likepeptide_associated[5e-32] |
| *Streptococcus* | *S. pyogenes* |  | MGAS10394 | AOI_1;orf021 | Unmodified | Mutacin_IV[4e-11] |
| *Streptococcus* | *S. pyogenes* |  | MGAS10394 | AOI_2;smallORF_3 | LAPs |  |
| *Streptococcus* | *S. pyogenes* |  | MGAS6180 | AOI_1;orf016 | Unmodified | Bacteriocin_like_peptide_associated[1e-30] |
| *Streptococcus* | *S. pyogenes* |  | MGAS6180 | AOI_1;orf017 | Unmodified | Blpi[4e-20] |
| *Streptococcus* | *S. pyogenes* |  | MGAS6180 | AOI_1;orf022 | Unmodified | Putative_bacteriocin[8e-51] |
| *Streptococcus* | *S. pyogenes* |  | MGAS6180 | AOI_1;orf023 | Unmodified | Bacteriocin_likepeptide_associated[6e-41] |
| *Streptococcus* | *S. pyogenes* |  | MGAS6180 | AOI_1;orf027 | Unmodified | Mutacin_IV[3e-07] |
| *Streptococcus* | *S. pyogenes* |  | MGAS6180 | AOI_2;smallORF_3 | LAPs |  |
| *Streptococcus* | *S. pyogenes* |  | MGAS6180 | AOI_3;smallORF_3 | Lanthipeptide_class_I | Streptin[8e-31] |
| *Streptococcus* | *S. pyogenes* |  | MGAS5005 | AOI_1;orf012 | Unmodified | Bacteriocin_like_peptide_associated[5e-52] |
| *Streptococcus* | *S. pyogenes* |  | MGAS5005 | AOI_1;orf013 | Unmodified | Blpi[4e-20] |
| *Streptococcus* | *S. pyogenes* |  | MGAS5005 | AOI_1;orf021 | Unmodified | Putative_bacteriocin[4e-27] |
| *Streptococcus* | *S. pyogenes* |  | MGAS5005 | AOI_1;orf022 | Unmodified | Bacteriocin_likepeptide_associated[4e-41] |
| *Streptococcus* | *S. pyogenes* |  | MGAS5005 | AOI_1;orf027 | Unmodified | Mutacin_IV[4e-11] |
| *Streptococcus* | *S. pyogenes* |  | MGAS5005 | AOI_2;smallORF_4 | LAPs |  |
| *Streptococcus* | *S. agalactiae* |  | A909 | AOI_1;orf007 | Bacteriocin >10kd | Zoocin_A[3e-117] |
| *Streptococcus* | *S. pyogenes* |  | MGAS9429 | AOI_1;orf016 | Unmodified | Putative_bacteriocin[1e-50] |
| *Streptococcus* | *S. pyogenes* |  | MGAS9429 | AOI_1;orf017 | Unmodified | Bacteriocin_likepeptide_associated[5e-32] |
| *Streptococcus* | *S. pyogenes* |  | MGAS9429 | AOI_1;orf021 | Unmodified | Mutacin_IV[4e-11] |
| *Streptococcus* | *S. pyogenes* |  | MGAS9429 | AOI_2;smallORF_4 | LAPs |  |
| *Streptococcus* | *S. pyogenes* |  | MGAS9429 | AOI_3;smallORF_3 | Lanthipeptide_class_I | Streptin[8e-31] |
| *Streptococcus* | *S. pyogenes* |  | MGAS10270 | AOI_1;orf013 | Unmodified | Bacteriocin_like_peptide_associated[7e-21] |
| *Streptococcus* | *S. pyogenes* |  | MGAS10270 | AOI_1;orf014 | Unmodified | Bacteriocin_like_peptide_associated[5e-10] |
| *Streptococcus* | *S. pyogenes* |  | MGAS10270 | AOI_1;orf015 | Unmodified | Blpi[4e-19] |
| *Streptococcus* | *S. pyogenes* |  | MGAS10270 | AOI_1;orf020 | Unmodified | Putative_bacteriocin[5e-50] |
| *Streptococcus* | *S. pyogenes* |  | MGAS10270 | AOI_1;orf021 | Unmodified | Bacteriocin_likepeptide_associated[1e-41] |
| *Streptococcus* | *S. pyogenes* |  | MGAS10270 | AOI_1;orf024 | Unmodified | Mutacin_IV[2e-06] |
| *Streptococcus* | *S. pyogenes* |  | MGAS10270 | AOI_2;orf008 | LAPs |  |
| *Streptococcus* | *S. pyogenes* |  | MGAS10270 | AOI_3;orf003 | Lanthipeptide_class_I | Streptin[8e-31] |
| *Streptococcus* | *S. pyogenes* |  | MGAS2096 | AOI_1;orf016 | Unmodified | Putative_bacteriocin[1e-50] |
| *Streptococcus* | *S. pyogenes* |  | MGAS2096 | AOI_1;orf017 | Unmodified | Bacteriocin_likepeptide_associated[4e-34] |
| *Streptococcus* | *S. pyogenes* |  | MGAS2096 | AOI_1;orf020 | Unmodified | Mutacin_IV[4e-11] |
| *Streptococcus* | *S. pyogenes* |  | MGAS2096 | AOI_2;smallORF_4 | LAPs |  |
| *Streptococcus* | *S. pyogenes* |  | MGAS2096 | AOI_3;smallORF_3 | Lanthipeptide_class_I | Streptin[8e-31] |
| *Streptococcus* | *S. pyogenes* |  | MGAS10750 | AOI_1;orf014 | Unmodified | Blpu[5e-18] |
| *Streptococcus* | *S. pyogenes* |  | MGAS10750 | AOI_1;orf019 | Unmodified | Blpo[7e-07] |
| *Streptococcus* | *S. pyogenes* |  | MGAS10750 | AOI_2;orf008 | Unmodified | Putative_bacteriocin[8e-51] |
| *Streptococcus* | *S. pyogenes* |  | MGAS10750 | AOI_2;orf009 | Unmodified | Bacteriocin_likepeptide_associated[6e-41] |
| *Streptococcus* | *S. pyogenes* |  | MGAS10750 | AOI_2;orf012 | Unmodified | Mutacin_IV[2e-06] |
| *Streptococcus* | *S. pyogenes* |  | MGAS10750 | AOI_3;smallORF_5 | LAPs |  |
| *Streptococcus* | *S. pyogenes* |  | MGAS10750 | AOI_4;orf004 | Lanthipeptide_class_I | Streptin[6e-30] |
| *Streptococcus* | *S. pneumoniae* |  | D39 | AOI_1;orf012 | Unmodified | Blpu[4e-31] |
| *Streptococcus* | *S. pneumoniae* |  | D39 | AOI_2;orf011 | Unmodified | Lactococcin_972_(Lcn972)[1e-08] |
| *Streptococcus* | *S. pneumoniae* |  | D39 | AOI_4;orf009 | Lanthipeptide_class_II |  |
| *Streptococcus* | *S. pyogenes* |  | Manfredo | AOI_2;orf019 | Unmodified | Mutacin_IV[4e-11] |
| *Streptococcus* | *S. pyogenes* |  | Manfredo | AOI_2;orf023 | Unmodified | Bacteriocin_likepeptide_associated[6e-41] |
| *Streptococcus* | *S. pyogenes* |  | Manfredo | AOI_2;orf024 | Unmodified | Putative_bacteriocin[8e-51] |
| *Streptococcus* | *S. pyogenes* |  | Manfredo | AOI_2;orf029 | Unmodified | Blpi[4e-20] |
| *Streptococcus* | *S. pyogenes* |  | Manfredo | AOI_2;orf030 | Unmodified | Bacteriocin_like_peptide_associated[5e-52] |
| *Streptococcus* | *S. suis* |  | 05ZYH33 | AOI_1;smallORF_10 | Lasso_peptide |  |
| *Streptococcus* | *S. gordonii* |  | Challis substr_CH1 | AOI_1;orf008 | Unmodified | Putative_bacteriocin[6e-33] |
| *Streptococcus* | *S. pneumoniae* |  | Hungary19A_6 | AOI_1;orf007 | Head_to_tail_cyclized_peptide | Blpu[4e-31] |
| *Streptococcus* | *S. pneumoniae* |  | Hungary19A_6 | AOI_1;orf015 | Head_to_tail_cyclized_peptide |  |
| *Streptococcus* | *S. pneumoniae* |  | Hungary19A_6 | AOI_1;smallORF_7 | Head_to_tail_cyclized_peptide | Blpu[4e-34] |
| *Streptococcus* | *S. pneumoniae* |  | Hungary19A_6 | AOI_1;smallORF_8 | Head_to_tail_cyclized_peptide | Blpu[3e-23] |
| *Streptococcus* | *S. pneumoniae* |  | Hungary19A_6 | AOI_2;orf011 | Unmodified | Lactococcin_972_(Lcn972)[7e-09] |
| *Streptococcus* | *S. pneumoniae* |  | Hungary19A_6 | AOI_3;orf009 | Unmodified | Blpi[4e-41] |
| *Streptococcus* | *S. pneumoniae* |  | Hungary19A_6 | AOI_3;orf010 | Unmodified | Blpj[5e-58] |
| *Streptococcus* | *S. pneumoniae* |  | Hungary19A_6 | AOI_3;orf012 | Unmodified | Blpk[6e-50] |
| *Streptococcus* | *S. pneumoniae* |  | Hungary19A_6 | AOI_4;orf011 | Unmodified | Lactococcin_972_(Lcn972)[4e-08] |
| *Streptococcus* | *S. pneumoniae* |  | Hungary19A_6 | AOI_5;orf008 | Unmodified | Lactococcin_972_(Lcn972)[6e-14] |
| *Streptococcus* | *S. pneumoniae* |  | CGSP14 | AOI_1;orf015 | Unmodified | Blpu[4e-31] |
| *Streptococcus* | *S. pneumoniae* |  | CGSP14 | AOI_2;orf009 | Lanthipeptide_class_I |  |
| *Streptococcus* | *S. pneumoniae* |  | CGSP14 | AOI_3;orf004 | Unmodified | Blpu[4e-30] |
| *Streptococcus* | *S. pneumoniae* |  | CGSP14 | AOI_3;orf012 | Unmodified | Blpm[2e-57] |
| *Streptococcus* | *S. pneumoniae* |  | CGSP14 | AOI_3;orf013 | Unmodified | Blpn[7e-34] |
| *Streptococcus* | *S. pneumoniae* |  | CGSP14 | AOI_3;orf015 | Unmodified | Blpo[7e-32] |
| *Streptococcus* | *S. pneumoniae* |  | CGSP14 | AOI_5;orf008 | Unmodified | Lactococcin_972_(Lcn972)[4e-13] |
| *Streptococcus* | *S. pneumoniae* |  | G54 | AOI_1;orf011 | Unmodified | Blpu[3e-46] |
| *Streptococcus* | *S. pneumoniae* |  | G54 | AOI_2;orf009 | Unmodified | Blpo[4e-09] |
| *Streptococcus* | *S. pneumoniae* |  | G54 | AOI_2;orf019 | Unmodified | Blpm[2e-52] |
| *Streptococcus* | *S. pneumoniae* |  | G54 | AOI_2;orf020 | Unmodified | Blpn[4e-33] |
| *Streptococcus* | *S. pneumoniae* |  | G54 | AOI_2;orf022 | Unmodified | Blpo[2e-32] |
| *Streptococcus* | *S. pneumoniae* |  | G54 | AOI_3;orf012 | Unmodified | Lactococcin_972_(Lcn972)[6e-11] |
| *Streptococcus* | *S. pneumoniae* |  | G54 | AOI_4;orf010 | Unmodified | Lactococcin_972_(Lcn972)[8e-14] |
| *Streptococcus* | *S. equi* | *zooepidemicus* | MGCS10565 | AOI_1;smallORF_3 | LAPs |  |
| *Streptococcus* | *S. equi* | *zooepidemicus* | MGCS10565 | AOI_2;orf013 | Unmodified | Blpu[3e-20] |
| *Streptococcus* | *S. equi* | *zooepidemicus* | MGCS10565 | AOI_3;orf013 | Unmodified | Blpn[2e-17] |
| *Streptococcus* | *S. equi* | *zooepidemicus* | MGCS10565 | AOI_3;orf014 | Unmodified | Blpm[7e-44] |
| *Streptococcus* | *S. equi* | *zooepidemicus* | MGCS10565 | AOI_3;orf018 | Unmodified | Ubericin-A[1e-28] |
| *Streptococcus* | *S. equi* | *zooepidemicus* | MGCS10565 | AOI_3;orf024 | Unmodified | Bovicin_255_peptide[3e-32] |
| *Streptococcus* | *S. equi* | *zooepidemicus* | MGCS10565 | AOI_4;orf012 | Unmodified | Thermophilin_13_chainb_(thmb)[2e-20] |
| *Streptococcus* | *S. equi* | *zooepidemicus* | MGCS10565 | AOI_4;orf013 | Unmodified | Thermophilin_A[2e-47] |
| *Streptococcus* | *S. equi* | *zooepidemicus* | MGCS10565 | AOI_5;orf017 | Unmodified | Putative_bacteriocin[2e-20] |
| *Streptococcus* | *S. pyogenes* |  | NZ131 | AOI_1;orf014 | Unmodified | Bacteriocin_like_peptide_associated[1e-21] |
| *Streptococcus* | *S. pyogenes* |  | NZ131 | AOI_1;orf015 | Unmodified | Bacteriocin_like_peptide_associated[5e-10] |
| *Streptococcus* | *S. pyogenes* |  | NZ131 | AOI_1;orf016 | Unmodified | Blpi[4e-20] |
| *Streptococcus* | *S. pyogenes* |  | NZ131 | AOI_1;orf023 | Unmodified | Putative_bacteriocin[4e-41] |
| *Streptococcus* | *S. pyogenes* |  | NZ131 | AOI_1;orf024 | Unmodified | Bacteriocin_likepeptide_associated[1e-41] |
| *Streptococcus* | *S. pyogenes* |  | NZ131 | AOI_1;orf028 | Unmodified | Mutacin_IV[4e-07] |
| *Streptococcus* | *S. pyogenes* |  | NZ131 | AOI_2;smallORF_3 | LAPs |  |
| *Streptococcus* | *S. pneumoniae* |  | ATCC_700669 | AOI_1;orf014 | Unmodified | Blpu[4e-31] |
| *Streptococcus* | *S. pneumoniae* |  | ATCC_700669 | AOI_2;orf008 | Unmodified | Blpi[8e-42] |
| *Streptococcus* | *S. pneumoniae* |  | ATCC_700669 | AOI_2;orf009 | Unmodified | Blpj[2e-49] |
| *Streptococcus* | *S. pneumoniae* |  | ATCC_700669 | AOI_2;orf010 | Unmodified | Blpn[5e-20] |
| *Streptococcus* | *S. pneumoniae* |  | ATCC_700669 | AOI_2;orf012 | Unmodified | Blpo[1e-31 |
| *Streptococcus* | *S. pneumoniae* |  | ATCC_700669 | AOI_2;orf013 | Unmodified | Blpu[5e-06] |
| *Streptococcus* | *S. pneumoniae* |  | ATCC_700669 | AOI_5;orf010 | Unmodified | Lactococcin_972_(Lcn972)[4e-13] |
| *Streptococcus* | *S. uberis* |  | 0140J | AOI_1;orf008 | Head_to_tail_cyclized_peptide | Uberolysin[1e-48] |
| *Streptococcus* | *S. uberis* |  | 0140J | AOI_2;orf007 | Unmodified | Blpo[6e-07] |
| *Streptococcus* | *S. uberis* |  | 0140J | AOI_2;orf010 | Unmodified | Putative_bacteriocin[2e-26] |
| *Streptococcus* | *S. uberis* |  | 0140J | AOI_2;orf012 | Unmodified | Bacteriocin_likepeptide_associated[6e-18] |
| *Streptococcus* | *S. uberis* |  | 0140J | AOI_2;orf015 | Unmodified | Bovicin_255_peptide[7e-28] |
| *Streptococcus* | *S. uberis* |  | 0140J | AOI_2;orf019 | Unmodified | Penocin_A[2e-13] |
| *Streptococcus* | *S. uberis* |  | 0140J | AOI_3;orf006 | Bacteriocin >10kd | Dysgalacticin[6e-84] |
| *Streptococcus* | *S. uberis* |  | JJA | AOI_1;orf006 | Head_to_tail_cyclized_peptide |  |
| *Streptococcus* | *S. uberis* |  | JJA | AOI_1;orf012 | Head_to_tail_cyclized_peptide |  |
| *Streptococcus* | *S. uberis* |  | JJA | AOI_1;smallORF_7 | Head_to_tail_cyclized_peptide |  |
| *Streptococcus* | *S. uberis* |  | JJA | AOI_1;smallORF_8 | Head_to_tail_cyclized_peptide |  |
| *Streptococcus* | *S. uberis* |  | JJA | AOI_2;orf012 | Unmodified | Lactococcin_972_(Lcn972)[1e-08] |
| *Streptococcus* | *S. uberis* |  | JJA | AOI_3;orf008 | Unmodified | Blpm[4e-55] |
| *Streptococcus* | *S. uberis* |  | JJA | AOI_3;orf009 | Unmodified | Blpn[1e-33] |
| *Streptococcus* | *S. uberis* |  | JJA | AOI_3;orf011 | Unmodified | Blpo[1e-31] |
| *Streptococcus* | *S. uberis* |  | JJA | AOI_3;orf012 | Unmodified | Blpu[7e-06] |
| *Streptococcus* | *S. uberis* |  | JJA | AOI_4;orf011 | Lanthipeptide_class_II |  |
| *Streptococcus* | *S. uberis* |  | JJA | AOI_5;orf009 | Unmodified | Lactococcin_972_(Lcn972)[9e-14] |
| *Streptococcus* | *S. pneumoniae* |  | P1031 | AOI_1;orf012 | Unmodified | Blpu[4e-31] |
| *Streptococcus* | *S. pneumoniae* |  | P1031 | AOI_2;orf010 | Unmodified | Lactococcin_972_(Lcn972)[1e-08] |
| *Streptococcus* | *S. pneumoniae* |  | P1031 | AOI_3;orf008 | Unmodified | Blpm[3e-57] |
| *Streptococcus* | *S. pneumoniae* |  | P1031 | AOI_3;orf009 | Unmodified | Blpn[1e-33] |
| *Streptococcus* | *S. pneumoniae* |  | P1031 | AOI_3;orf011 | Unmodified | Blpo[1e-31] |
| *Streptococcus* | *S. pneumoniae* |  | P1031 | AOI_3;orf012 | Unmodified | Blpu[7e-06] |
| *Streptococcus* | *S. pneumoniae* |  | P1031 | AOI_4;orf012 | Unmodified | Lactococcin_972_(Lcn972)[5e-08] |
| *Streptococcus* | *S. pneumoniae* |  | 70585 | AOI_1;orf006 | Head_to_tail_cyclized_peptide | Blpu[4e-46] |
| *Streptococcus* | *S. pneumoniae* |  | 70585 | AOI_1;orf012 | Head_to_tail_cyclized_peptide |  |
| *Streptococcus* | *S. pneumoniae* |  | 70585 | AOI_1;smallORF_7 | Head_to_tail_cyclized_peptide | Blpu[3e-50] |
| *Streptococcus* | *S. pneumoniae* |  | 70585 | AOI_1;smallORF_8 | Head_to_tail_cyclized_peptide | Blpu[2e-37] |
| *Streptococcus* | *S. pneumoniae* |  | 70585 | AOI_2;orf009 | Unmodified | Blpi[8e-42] |
| *Streptococcus* | *S. pneumoniae* |  | 70585 | AOI_2;orf010 | Unmodified | Blpj[5e-58] |
| *Streptococcus* | *S. pneumoniae* |  | 70585 | AOI_2;orf011 | Unmodified | Blpk[6e-50] |
| *Streptococcus* | *S. pneumoniae* |  | 70585 | AOI_2;orf023 | Unmodified | Blpm[2e-57] |
| *Streptococcus* | *S. pneumoniae* |  | 70585 | AOI_2;orf024 | Unmodified | Blpn[3e-33] |
| *Streptococcus* | *S. pneumoniae* |  | 70585 | AOI_2;orf025 | Unmodified | Blpo[2e-32] |
| *Streptococcus* | *S. pneumoniae* |  | 70585 | AOI_3;orf009 | Unmodified | Lactococcin_972_(Lcn972)[6e-11] |
| *Streptococcus* | *S. pneumoniae* |  | 70585 | AOI_4;smallORF_5 | Lasso_peptide |  |
| *Streptococcus* | *S. pneumoniae* |  | 70585 | AOI_5;orf009 | Unmodified | Lactococcin_972_(Lcn972)[9e-14] |
| *Streptococcus* | *S. pneumoniae* |  | Taiwan19F_14 | AOI_1;orf010 | Unmodified | Blpu[2e-34] |
| *Streptococcus* | *S. pneumoniae* |  | Taiwan19F_14 | AOI_2;orf010 | Unmodified | Lactococcin_972_(Lcn972)[1e-08] |
| *Streptococcus* | *S. pneumoniae* |  | Taiwan19F_14 | AOI_3;orf011 | Unmodified | Blpo[2e-12] |
| *Streptococcus* | *S. pneumoniae* |  | Taiwan19F_14 | AOI_3;orf012 | Unmodified | Acidocin_LF221B(gassericink7b)[3e-11] |
| *Streptococcus* | *S. pneumoniae* |  | Taiwan19F_14 | AOI_3;orf023 | Unmodified | Blpm[6e-24] |
| *Streptococcus* | *S. pneumoniae* |  | Taiwan19F_14 | AOI_4;orf010 | Unmodified | Lactococcin_972_(Lcn972)[3e-09] |
| *Streptococcus* | *S. equi* | *zooepidemicus* |  | AOI_1;orf020 | Unmodified | Putative_bacteriocin[7e-20] |
| *Streptococcus* | *S. equi* | *zooepidemicus* |  | AOI_2;orf007 | Unmodified | Thermophilin_A[2e-47] |
| *Streptococcus* | *S. equi* | *zooepidemicus* |  | AOI_2;orf008 | Unmodified | Thermophilin_13_chainb_(thmb)[2e-20] |
| *Streptococcus* | *S. equi* | *zooepidemicus* |  | AOI_3;smallORF_3 | Lanthipeptide_class_II | Streptococcin_A-FF22[5e-27] |
| *Streptococcus* | *S. equi* | *zooepidemicus* |  | AOI_3;smallORF_4 | Lanthipeptide_class_II | Streptococcin_A-FF22[1e-28] |
| *Streptococcus* | *S. equi* | *zooepidemicus* |  | AOI_3;smallORF_6 | Lanthipeptide_class_II | Mcda1[5e-25] |
| *Streptococcus* | *S. equi* | *zooepidemicus* |  | AOI_4;orf010 | Unmodified | Blpu[1e-14] |
| *Streptococcus* | *S. equi* | *Equi* | 4047 | AOI_1;smallORF_2 | LAPs |  |
| *Streptococcus* | *S. equi* | *Equi* | 4047 | AOI_2;orf007 | Unmodified | Putative_bacteriocin[2e-11] |
| *Streptococcus* | *S. equi* | *Equi* | 4047 | AOI_3;orf015 | Unmodified | Penocin_A[1e-13] |
| *Streptococcus* | *S. equi* | *Equi* | 4047 | AOI_4;orf013 | Unmodified | Blpn[7e-17] |
| *Streptococcus* | *S. equi* | *Equi* | 4047 | AOI_4;orf014 | Unmodified | Blpm[2e-41] |
| *Streptococcus* | *S. equi* | *Equi* | 4047 | AOI_4;orf20 | Unmodified | Bovicin_255_peptide[9e-34] |
| *Streptococcus* | *S. equi* | *Equi* | 4047 | AOI_5;orf014 | Unmodified | Putative_bacteriocin[5e-14] |
| *Streptococcus* | *S. dysgalactiae* | *equisimilis* | GGS_124 | AOI_1;orf009 | Unmodified | Blpu[3e-09] |
| *Streptococcus* | *S. dysgalactiae* | *equisimilis* | GGS_124 | AOI_1;orf013 | Unmodified | Blpm[1e-32] |
| *Streptococcus* | *S. dysgalactiae* | *equisimilis* | GGS_124 | AOI_2;smallORF_6 | LAPs |  |
| *Streptococcus* | *S. dysgalactiae* | *equisimilis* | GGS_124 | AOI_3;orf010 | Unmodified | Ubericin-A[4e-25] |
| *Streptococcus* | *S. suis* |  | SC84 | AOI_1;smallORF_9 | Lasso_peptide |  |
| *Streptococcus* | *S. suis* |  | BM407 | AOI_1;orf011 | Unmodified | Thermophilin_A[6e-11] |
| *Streptococcus* | *S. suis* |  | BM407 | AOI_2;smallORF_3 | Lasso_peptide |  |
| *Streptococcus* | *S. gallolyticus* |  | UCN34 | AOI_1;orf013 | Unmodified | Blpi[1e-23] |
| *Streptococcus* | *S. gallolyticus* |  | UCN34 | AOI_1;orf015 | Unmodified | Thermophilin_A[9e-21] |
| *Streptococcus* | *S. gallolyticus* |  | UCN34 | AOI_2;smallORF_5 | LAPs |  |
| *Streptococcus* | *S. mutans* |  | NN2025 | AOI_1;orf004 | Unmodified | Blpk[1e-06] |
| *Streptococcus* | *S. mutans* |  | NN2025 | AOI_1;orf007 | Unmodified | Bovicin_255_peptide[5e-08] |
| *Streptococcus* | *S. mutans* |  | NN2025 | AOI_1;orf009 | Unmodified | Thermophilin_A[2e-12] |
| *Streptococcus* | *S. mutans* |  | NN2025 | AOI_2;orf009 | Unmodified | Streptococcin_A-FF22[4e-20] |
| *Streptococcus* | *S. mutans* |  | NN2025 | AOI_2;orf011 | Lanthipeptide_class_II | Streptococcin_A-FF22[1e-13] |
| *Streptococcus* | *S. mutans* |  | NN2025 | AOI_2;orf012 | Lanthipeptide_class_II | Streptococcin_A-FF22[2e-21] |
| *Streptococcus* | *S. mutans* |  | NN2025 | AOI_2;orf013 | Lanthipeptide_class_II | Mcda1[4e-22] |
| *Streptococcus* | *S. mutans* |  | NN2025 | AOI_3;orf009 | Unmodified | Putative_bacteriocin[1e-47] |
| *Streptococcus* | *S. pneumoniae* |  | TCH8431_19A | AOI_1;orf010 | Unmodified | Blpu[4e-46] |
| *Streptococcus* | *S. pneumoniae* |  | TCH8431_19A | AOI_2;orf009 | Unmodified | Lactococcin_972_(Lcn972)[1e-08] |
| *Streptococcus* | *S. pneumoniae* |  | TCH8431_19A | AOI_3;orf013 | Unmodified | Blpo[2e-12] |
| *Streptococcus* | *S. pneumoniae* |  | TCH8431_19A | AOI_3;orf014 | Unmodified | Acidocin_LF221B(gassericink7b)[3e-11] |
| *Streptococcus* | *S. pneumoniae* |  | TCH8431_19A | AOI_3;orf025 | Unmodified | Blpm[6e-24] |
| *Streptococcus* | *S. pneumoniae* |  | TCH8431_19A | AOI_4;orf010 | Unmodified | Lactococcin_972_(Lcn972)[3e-09] |
| *Streptococcus* | *S. pneumoniae* |  | AP200 | AOI_1;orf009 | Unmodified | Blpm[3e-57] |
| *Streptococcus* | *S. pneumoniae* |  | AP200 | AOI_1;orf010 | Unmodified | Blpn[1e-33] |
| *Streptococcus* | *S. pneumoniae* |  | AP200 | AOI_1;orf012 | Unmodified | Blpo[1e-31] |
| *Streptococcus* | *S. pneumoniae* |  | AP200 | AOI_2;orf009 | Unmodified | Lactococcin_972_(Lcn972)[9e-14] |
| *Streptococcus* | *S. pneumoniae* |  | 670_6B | AOI_1;orf011 | Unmodified | Blpu[4e-31] |
| *Streptococcus* | *S. pneumoniae* |  | 670_6B | AOI_2;orf010 | Unmodified | Blpi[3e-09] |
| *Streptococcus* | *S. pneumoniae* |  | 670_6B | AOI_2;orf021 | Unmodified | Blpm[1e-56] |
| *Streptococcus* | *S. pneumoniae* |  | 670_6B | AOI_2;orf022 | Unmodified | Blpn[7e-34] |
| *Streptococcus* | *S. pneumoniae* |  | 670_6B | AOI_2;orf024 | Unmodified | Blpo[5e-10] |
| *Streptococcus* | *S. pneumoniae* |  | 670_6B | AOI_3;orf011 | Unmodified | Lactococcin_972_(Lcn972)[4e-08] |
| *Streptococcus* | *S. pneumoniae* |  | 670_6B | AOI_4;orf009 | Unmodified | Lactococcin_972_(Lcn972)[9e-14] |
| *Streptococcus* | *S. gallolyticus* | *gallolyticus* | ATCC_BAA_2069 | AOI_2;orf010 | Glycocin |  |
| *Streptococcus* | *S. gallolyticus* | *gallolyticus* | ATCC_BAA_2069 | AOI_2;orf020 | Glycocin |  |
| *Streptococcus* | *S. gallolyticus* | *gallolyticus* | ATCC_BAA_2069 | AOI_2;orf024 | Glycocin | Blpi[1e-23] |
| *Streptococcus* | *S. gallolyticus* | *gallolyticus* | ATCC_BAA_2069 | AOI_2;orf026 | Glycocin | Thermophilin_A[9e-21] |
| *Streptococcus* | *S. gallolyticus* | *gallolyticus* | ATCC_BAA_2069 | AOI_2;smallORF_4 | Glycocin |  |
| *Streptococcus* | *S. gallolyticus* | *gallolyticus* | ATCC_BAA_2069 | AOI_3;smallORF_5 | LAPs |  |
| *Streptococcus* | *S. oralis* |  | Uo5 | AOI_1;orf005 | Unmodified | Lactococcin_972_(Lcn972)[2e-09] |
| *Streptococcus* | *S. pasteurianus* |  | ATCC_43144 | AOI_2;orf010 | Unmodified | Bovicin_255_peptide[2e-10] |
| *Streptococcus* | *S. salivarius* |  | CCHSS3 | AOI_4;orf005 | Unmodified | Blpk[5e-12] |
| *Streptococcus* | *S. salivarius* |  | CCHSS3 | AOI_4;orf015 | Unmodified | Blpu[8e-29] |
| *Streptococcus* | *S. salivarius* |  | CCHSS3 | AOI_3;orf006 | Unmodified | Lactococcin_972_(Lcn972)[5e-14 |
| *Streptococcus* | *S. salivarius* |  | CCHSS3 | AOI_2;orf012 | Lanthipeptide_class_II | Salivaricin_9[5e-37] |
| *Streptococcus* | *S. salivarius* |  | CCHSS3 | AOI_1;orf012 | Unmodified | Enterocin_X_chain_beta[1e-10] |
| *Streptococcus* | *S. pseudopneumoniae* |  | IS7493 | AOI_1;orf013 | Unmodified | Pf10439.4 |
| *Streptococcus* | *S. pseudopneumoniae* |  | IS7493 | AOI_1;orf014 | Unmodified | Blpu[2e-28] |
| *Streptococcus* | *S. pseudopneumoniae* |  | IS7493 | AOI_2;orf001 | Head_to_tail_cyclized_peptide |  |
| *Streptococcus* | *S. pseudopneumoniae* |  | IS7493 | AOI_2;orf012 | Head_to_tail_cyclized_peptide |  |
| *Streptococcus* | *S. pseudopneumoniae* |  | IS7493 | AOI_3;orf009 | Unmodified | Lactococcin_972_(Lcn972)[1e-08] |
| *Streptococcus* | *S. pseudopneumoniae* |  | IS7493 | AOI_4;orf010 | Unmodified | Lactococcin_972_(Lcn972)[1e-10] |
| *Streptococcus* | *S. pseudopneumoniae* |  | IS7493 | AOI_5;orf020 | Unmodified | Mutacin_IV[8e-17] |
| *Streptococcus* | *S. pseudopneumoniae* |  | IS7493 | AOI_5;orf022 | Unmodified | Blpo[8e-11] |
| *Streptococcus* | *S. pseudopneumoniae* |  | IS7493 | AOI_6;orf009 | Unmodified | Lactococcin_972_(Lcn972)[1e-09] |
| *Streptococcus* | *S. macedonicus* |  | ACA_DC_198 | AOI_1;orf008 | Lanthipeptide_class_II | Mcda1[1e-35] |
| *Streptococcus* | *S. macedonicus* |  | ACA_DC_198 | AOI_1;orf009 | Lanthipeptide_class_II | Macedocin[3e-34] |
| *Streptococcus* | *S. macedonicus* |  | ACA_DC_198 | AOI_1;orf010 | Lanthipeptide_class_II | Macedocin[3e-34] |
| *Streptococcus* | *S. macedonicus* |  | ACA_DC_198 | AOI_2;smallORF_4 | Lanthipeptide_class_II |  |
| *Streptococcus* | *S. macedonicus* |  | ACA_DC_198 | AOI_3;orf011 | Unmodified | Blpi[3e-13] |
| *Streptococcus* | *S. macedonicus* |  | ACA_DC_198 | AOI_3;orf013 | Unmodified | Thermophilin_A[3e-17] |
| *Streptococcus* | *S. macedonicus* |  | ACA_DC_198 | AOI_3;orf018 | Unmodified | Bovicin_255_peptide[2e-10] |
| *Streptococcus* | *S. infantarius* | *infantarius* | CJ18 | AOI_1;orf011 | Unmodified | Bovicin_255_peptide[7e-09] |
| *Streptococcus* | *S. infantarius* | *infantarius* | CJ18 | AOI_1;orf013 | Unmodified | Bovicin_255_peptide[1e-09] |
| *Streptococcus* | *S. infantarius* | *infantarius* | CJ18 | AOI_1;orf015 | Unmodified | Bovicin_255_peptide[5e-10] |
| *Streptococcus* | *S. infantarius* | *infantarius* | CJ18 | AOI_1;orf019 | Unmodified | Bovicin_255_peptide[8e-49] |
| *Streptococcus* | *S. pyogenes* |  | MGAS15252 | AOI_1;orf010 | Unmodified | Bacteriocin_like_peptide_associated[5e-52] |
| *Streptococcus* | *S. pyogenes* |  | MGAS15252 | AOI_1;orf011 | Unmodified | Blpi[4e-20] |
| *Streptococcus* | *S. pyogenes* |  | MGAS15252 | AOI_1;orf017 | Unmodified | Putative_bacteriocin[4e-41] |
| *Streptococcus* | *S. pyogenes* |  | MGAS15252 | AOI_1;orf018 | Unmodified | Bacteriocin_likepeptide_associated[1e-41] |
| *Streptococcus* | *S. pyogenes* |  | MGAS15252 | AOI_1;orf023 | Unmodified | Mutacin_IV[3e-07 |
| *Streptococcus* | *S. pyogenes* |  | MGAS15252 | AOI_2;smallORF_3 | LAPs |  |
| *Streptococcus* | *S. pyogenes* |  | MGAS1882 | AOI_1;orf014 | Unmodified | Bacteriocin_like_peptide_associated[5e-52] |
| *Streptococcus* | *S. pyogenes* |  | MGAS1882 | AOI_1;orf015 | Unmodified | Blpi[4e-20] |
| *Streptococcus* | *S. pyogenes* |  | MGAS1882 | AOI_1;orf020 | Unmodified | Putative_bacteriocin[4e-41] |
| *Streptococcus* | *S. pyogenes* |  | MGAS1882 | AOI_1;orf021 | Unmodified | Bacteriocin_likepeptide_associated[1e-41] |
| *Streptococcus* | *S. pyogenes* |  | MGAS1882 | AOI_1;orf025 | Unmodified | Mutacin_IV[3e-07] |
| *Streptococcus* | *S. pyogenes* |  | MGAS1882 | AOI_2;smallORF_3 | LAPs |  |
| *Streptococcus* | *S. dysgalactiae* | *equisimilis* | ATCC_12394 | AOI_1;smallORF_2 | Lanthipeptide_class_I |  |
| *Streptococcus* | *S. dysgalactiae* | *equisimilis* | ATCC_12394 | AOI_2;orf014 | Unmodified | Blpu[1e-09] |
| *Streptococcus* | *S. dysgalactiae* | *equisimilis* | ATCC_12394 | AOI_2;orf020 | Unmodified | Blpm[3e-33] |
| *Streptococcus* | *S. dysgalactiae* | *equisimilis* | ATCC_12394 | AOI_3;smallORF_6 | LAPs |  |
| *Streptococcus* | *S. gallolyticus* | *gallolyticus* | ATCC_43143 | AOI_1;orf011 | Unmodified | Bovicin_255_peptide[2e-06] |
| *Streptococcus* | *S. gallolyticus* | *gallolyticus* | ATCC_43143 | AOI_1;orf012 | Unmodified | Blpi[1e-23] |
| *Streptococcus* | *S. gallolyticus* | *gallolyticus* | ATCC_43143 | AOI_1;orf014 | Unmodified | Thermophilin_A[9e-21] |
| *Streptococcus* | *S. gallolyticus* | *gallolyticus* | ATCC_43143 | AOI_2;smallORF_5 | LAPs |  |
| *Streptococcus* | *S. equi* | *zooepidemicus* | ATCC_35246 | AOI_1;smallORF_2 | LAPs |  |
| *Streptococcus* | *S. equi* | *zooepidemicus* | ATCC_35246 | AOI_2;orf010 | Unmodified | Blpu[1e-14] |
| *Streptococcus* | *S. equi* | *zooepidemicus* | ATCC_35246 | AOI_3;orf013 | Unmodified | Blpm[6e-42] |
| *Streptococcus* | *S. equi* | *zooepidemicus* | ATCC_35246 | AOI_3;orf019 | Unmodified | Bovicin_255_peptide[8e-34] |
| *Streptococcus* | *S. equi* | *zooepidemicus* | ATCC_35246 | AOI_4;orf016 | Unmodified | Bacteriocin_likepeptide_associated[2e-15] |
| *Streptococcus* | *S. equi* | *zooepidemicus* | ATCC_35246 | AOI_4;orf018 | Unmodified | Putative_bacteriocin[1e-23] |
| *Streptococcus* | *S. pneumoniae* |  | INV104 | AOI_1;orf014 | Unmodified | Blpu[4e-31] |
| *Streptococcus* | *S. pneumoniae* |  | INV104 | AOI_3;orf001 | Unmodified | Blpi[8e-42] |
| *Streptococcus* | *S. pneumoniae* |  | INV104 | AOI_3;orf003 | Unmodified | Blpj[1e-54] |
| *Streptococcus* | *S. pneumoniae* |  | INV104 | AOI_3;orf004 | Unmodified | Blpk[2e-49] |
| *Streptococcus* | *S. pneumoniae* |  | INV104 | AOI_3;orf010 | Unmodified | Blpm[2e-57] |
| *Streptococcus* | *S. pneumoniae* |  | INV104 | AOI_3;orf011 | Unmodified | Blpn[7e-34] |
| *Streptococcus* | *S. pneumoniae* |  | INV104 | AOI_3;orf013 | Unmodified | Blpo[2e-32] |
| *Streptococcus* | *S. pneumoniae* |  | OXC141 | AOI_1;orf013 | Unmodified | Blpi[4e-41] |
| *Streptococcus* | *S. pneumoniae* |  | OXC141 | AOI_1;orf014 | Unmodified | Blpo[4e-31] |
| *Streptococcus* | *S. pneumoniae* |  | OXC141 | AOI_2;orf010 | Unmodified | Lactococcin_972_(Lcn972)[6e-09] |
| *Streptococcus* | *S. pneumoniae* |  | OXC141 | AOI_3;orf008 | Unmodified | Lactococcin_972_(Lcn972)[9e-14] |
| *Streptococcus* | *S. pneumoniae* |  | INV200 | AOI_1;orf014 | Unmodified | Blpu[4e-31] |
| *Streptococcus* | *S. pneumoniae* |  | INV200 | AOI_2;orf007 | Lanthipeptide_class_I |  |
| *Streptococcus* | *S. pneumoniae* |  | INV200 | AOI_3;orf004 | Unmodified | Blpu[4e-30] |
| *Streptococcus* | *S. pneumoniae* |  | INV200 | AOI_3;orf012 | Unmodified | Blpm[2e-57] |
| *Streptococcus* | *S. pneumoniae* |  | INV200 | AOI_3;orf013 | Unmodified | Blpn[7e-34] |
| *Streptococcus* | *S. pneumoniae* |  | INV200 | AOI_3;orf014 | Unmodified | Blpo[7e-32] |
| *Streptococcus* | *S. pneumoniae* |  | INV200 | AOI_4;orf012 | Unmodified | Lactococcin_972_(Lcn972)[5e-08] |
| *Streptococcus* | *S. pneumoniae* |  | INV200 | AOI_6;orf009 | Unmodified | Lactococcin_972_(Lcn972)[4e-13] |
| *Streptococcus* | *S. salivarius* |  | 57I | AOI_1;orf007 | Unmodified | Blpu[9e-12] |
| *Streptococcus* | *S. salivarius* |  | 57I | AOI_2;orf002 | Unmodified | Blpu[5e-31] |
| *Streptococcus* | *S. salivarius* |  | 57I | AOI_2;orf012 | Unmodified | Blpk[5e-12] |
| *Streptococcus* | *S. salivarius* |  | 57I | AOI_3;orf005 | Unmodified | Enterocin_X_chain_beta[7e-10] |
| *Streptococcus* | *S. salivarius* |  | JIM8777 | AOI_1;orf010 | Unmodified | Blpk[8e-14] |
| *Streptococcus* | *S. salivarius* |  | JIM8777 | AOI_1;orf019 | Unmodified | Blpu[1e-31] |
| *Streptococcus* | *S. salivarius* |  | JIM8777 | AOI_2;orf012 | Unmodified | Blpu[1e-11] |
| *Streptococcus* | *S. pyogenes* |  | Alab49 | AOI_1;orf013 | Unmodified | Blpu[1e-14] |
| *Streptococcus* | *S. pyogenes* |  | Alab49 | AOI_1;orf018 | Unmodified | Blpo[7e-07] |
| *Streptococcus* | *S. pyogenes* |  | Alab49 | AOI_2;orf007 | Unmodified | Putative_bacteriocin[1e-12] |
| *Streptococcus* | *S. pyogenes* |  | Alab49 | AOI_2;orf013 | Unmodified | Bacteriocin_like_peptide_associated[5e-52] |
| *Streptococcus* | *S. pyogenes* |  | Alab49 | AOI_2;orf014 | Unmodified | Blpi[4e-20] |
| *Streptococcus* | *S. pyogenes* |  | Alab49 | AOI_2;orf022 | Unmodified | Putative_bacteriocin[4e-41] |
| *Streptococcus* | *S. pyogenes* |  | Alab49 | AOI_2;orf023 | Unmodified | Bacteriocin_likepeptide_associated[1e-41] |
| *Streptococcus* | *S. pyogenes* |  | Alab49 | AOI_2;orf026 | Unmodified | Mutacin_IV[3e-07] |
| *Streptococcus* | *S. pyogenes* |  | Alab49 | AOI_3;smallORF_3 | LAPs |  |
| *Streptococcus* | *S. pyogenes* |  | Alab49 | AOI_4;orf013 | Lanthipeptide_class_II | Putative_lantibiotic[2e-32] |
| *Streptococcus* | *S. suis* |  | JS14 | AOI_1;smallORF_2 | Lanthipeptide_class_I | Nisin_U[3e-35] |
| *Streptococcus* | *S. suis* |  | D12 | AOI_1;smallORF_8 | Lanthipeptide_class_II |  |
| *Streptococcus* | *S. suis* |  | D12 | AOI_2;orf012 | Unmodified | Lactococcin_972_(Lcn972)[4e-11] |
| *Streptococcus* | *S. mutans* |  | LJ23 | AOI_1;orf011 | Unmodified | Blpk[2e-09] |
| *Streptococcus* | *S. mutans* |  | LJ23 | AOI_2;orf010 | Unmodified | Putative_bacteriocin[6e-51] |
| *Streptococcus* | *S. pneumoniae* |  | ST556 | AOI_1;orf012 | Unmodified | Blpu[2e-34] |
| *Streptococcus* | *S. pneumoniae* |  | ST556 | AOI_2;orf009 | Unmodified | Lactococcin_972_(Lcn972)[1e-08] |
| *Streptococcus* | *S. pneumoniae* |  | ST556 | AOI_3;orf012 | Unmodified | Blpo[2e-12] |
| *Streptococcus* | *S. pneumoniae* |  | ST556 | AOI_3;orf013 | Unmodified | Acidocin_LF221B(gassericink7b)[3e-11] |
| *Streptococcus* | *S. pneumoniae* |  | ST556 | AOI_3;orf020 | Unmodified | Blpu[8e-07] |
| *Streptococcus* | *S. pneumoniae* |  | ST556 | AOI_3;orf026 | Unmodified | Blpm[6e-24] |
| *Streptococcus* | *S. pneumoniae* |  | ST556 | AOI_4;orf011 | Unmodified | Lactococcin_972_(Lcn972)[3e-09] |
| *Streptococcus* | *S. parasanguinis* |  | FW213 | AOI_1;orf006 | Unmodified | Lactococcin_972_(Lcn972)[5e-10] |
| *Streptococcus* | *S. intermedius* |  | JTH08 | AOI_1;orf008 | Unmodified | Blpu[2e-08] |
| *Streptococcus* | *S. intermedius* |  | JTH08 | AOI_1;orf011 | Unmodified | Hiracin_JM79[2e-11] |
| *Streptococcus* | *S. intermedius* |  | JTH08 | AOI_1;orf015 | Unmodified | Blpu[3e-07] |
| *Streptococcus* | *S. intermedius* |  | JTH08 | AOI_1;orf017 | Unmodified | Mutacin_IV[5e-07] |
| *Streptococcus* | *S. intermedius* |  | JTH08 | AOI_1;orf019 | Unmodified | Blpu[6e-26] |
| *Streptococcus* | *S. mutans* |  | GS_5 | AOI_1;orf008 | Unmodified | Mutacin_IV[1e-49] |
| *Streptococcus* | *S. mutans* |  | GS_5 | AOI_1;orf009 | Unmodified | Bovicin_255_peptide[1e-08] |
| *Streptococcus* | *S. mutans* |  | GS_5 | AOI_2;orf010 | Unmodified | Putative_bacteriocin[6e-51] |
| *Streptococcus* | *S. mutans* |  | GS_5 | AOI_3;orf012 | Unmodified | Thermophilin_A[9e-09] |
| *Streptococcus* | *S. mutans* |  | GS_5 | AOI_3;orf013 | Unmodified | Thermophilin_A[6e-08] |
| *Streptococcus* | *S. mutans* |  | GS_5 | AOI_3;orf023 | Unmodified | Blpk[1e-06] |
| *Streptococcus* | *S. mutans* |  | GS_5 | AOI_4;orf012 | Lanthipeptide_class_II | Smbb[2e-42] |
| *Streptococcus* | *S. mutans* |  | GS_5 | AOI_4;smallORF_4 | Lanthipeptide_class_II | Bhta2[1e-39] |
| *Streptococcus* | *S. mutans* |  | GS_5 | AOI_4;smallORF_5 | Lanthipeptide_class_II | Bhta2[2e-28] |
| *Streptococcus* | *S. pneumoniae* |  | SPNA45 | AOI_1;orf014 | Unmodified | Blpu[2e-29] |
| *Streptococcus* | *S. pneumoniae* |  | SPNA45 | AOI_2;orf010 | Unmodified | Lactococcin_972_(Lcn972)[1e-08] |
| *Streptococcus* | *S. pneumoniae* |  | SPNA45 | AOI_3;orf007 | Unmodified | Blpu[7e-19] |
| *Streptococcus* | *S. pneumoniae* |  | gamPNI0373 | AOI_1;orf011 | Unmodified | Blpu[4e-31] |
| *Streptococcus* | *S. pneumoniae* |  | gamPNI0373 | AOI_2;orf011 | Unmodified | Lactococcin_972_(Lcn972)[9e-09] |
| *Streptococcus* | *S. pneumoniae* |  | gamPNI0373 | AOI_3;orf008 | Unmodified | Blpm[3e-57] |
| *Streptococcus* | *S. pneumoniae* |  | gamPNI0373 | AOI_3;orf009 | Unmodified | Blpn[1e-33] |
| *Streptococcus* | *S. pneumoniae* |  | gamPNI0373 | AOI_3;orf011 | Unmodified | Blpo[1e-31] |
| *Streptococcus* | *S. pneumoniae* |  | gamPNI0373 | AOI_3;orf012 | Unmodified | Blpu[7e-06] |
| *Streptococcus* | *S. pneumoniae* |  | gamPNI0373 | AOI_4;orf009 | Unmodified | Lactococcin_972_(Lcn972)[6e-11] |
| *Streptococcus* | *S. agalactiae* |  | GD201008_001 | AOI_1;orf006 | Bacteriocin >10kd | Zoocin_A[3e-117] |
| *Streptococcus* | *S. dysgalactiae* | *equisimilis* | RE378 | AOI_3;orf009 | Unmodified | Ubericin-A[4e-25] |
| *Streptococcus* | *S. dysgalactiae* | *equisimilis* | RE378 | AOI_2;smallORF_5 | LAPs |  |
| *Streptococcus* | *S. dysgalactiae* | *equisimilis* | RE378 | AOI_1;orf011 | Unmodified | Bacteriocin_like_peptide_associated[5e-52] |
| *Streptococcus* | *S. dysgalactiae* | *equisimilis* | RE378 | AOI_1;orf012 | Unmodified | Blpi[4e-20] |
| *Streptococcus* | *S. dysgalactiae* | *equisimilis* | RE378 | AOI_1;orf017 | Unmodified | Putative_bacteriocin[4e-40] |
| *Streptococcus* | *S. dysgalactiae* | *equisimilis* | RE378 | AOI_1;orf018 | Unmodified | Bacteriocin_likepeptide_associated[2e-31] |
| *Streptococcus* | *S. dysgalactiae* | *equisimilis* | RE378 | AOI_1;orf021 | Unmodified | Mutacin_IV[4e-11] |
| *Streptococcus* | *S. pyogenes* |  | A20 | AOI_1;orf013 | Unmodified | Bacteriocin_like_peptide_associated[5e-52] |
| *Streptococcus* | *S. pyogenes* |  | A20 | AOI_1;orf014 | Unmodified | Blpi[4e-20] |
| *Streptococcus* | *S. pyogenes* |  | A20 | AOI_1;orf021 | Unmodified | Putative_bacteriocin[4e-27] |
| *Streptococcus* | *S. pyogenes* |  | A20 | AOI_1;orf022 | Unmodified | Bacteriocin_likepeptide_associated[4e-41] |
| *Streptococcus* | *S. pyogenes* |  | A20 | AOI_1;orf027 | Unmodified | Mutacin_IV[4e-11] |
| *Streptococcus* | *S. pyogenes* |  | A20 | AOI_2;smallORF_4 | LAPs |  |
| *Streptococcus* | *S. dysgalactiae* | *equisimilis* | AC_2713 | AOI_1;orf007 | Lanthipeptide_class_II | Putative_lantibiotic[8e-32] |
| *Streptococcus* | *S. dysgalactiae* | *equisimilis* | AC_2713 | AOI_1;smallORF_3 | Lanthipeptide_class_II | Salivaricin_A2[1e-31] |
| *Streptococcus* | *S. dysgalactiae* | *equisimilis* | AC_2713 | AOI_2;orf012 | Unmodified | Blpu[3e-09] |
| *Streptococcus* | *S. dysgalactiae* | *equisimilis* | AC_2713 | AOI_2;orf018 | Unmodified | Blpm[3e-33] |
| *Streptococcus* | *S. dysgalactiae* | *equisimilis* | AC_2713 | AOI_3;smallORF_7 | LAPs |  |
| *Streptococcus* | *S. dysgalactiae* | *equisimilis* | AC_2713 | AOI_4;orf010 | Unmodified | Ubericin-A[7e-24] |
| *Streptococcus* | *S. agalactiae* |  | SA20_06 | AOI_2;orf015 | Unmodified | Blpm[9e-33] |
| *Streptococcus* | *S. agalactiae* |  | SA20_06 | AOI_2;orf016 | Unmodified | Blpn[2e-17] |
| *Streptococcus* | *S. agalactiae* |  | SA20_06 | AOI_1;orf006 | Bacteriocin >10kd | Zoocin_A[1e-115] |
| *Streptococcus* | *S. suis* |  | SC070731 | AOI_1;smallORF_2 | Lanthipeptide_class_I | Nisin_U[3e-35] |
| *Streptococcus* | *S. pyogenes* |  | M1_476 | AOI_2;smallORF_4 | LAPs |  |
| *Streptococcus* | *S. pyogenes* |  | M1_476 | AOI_1;orf008 | Unmodified | Bacteriocin_like_peptide_associated[5e-52] |
| *Streptococcus* | *S. pyogenes* |  | M1_476 | AOI_1;orf009 | Unmodified | Blpi[4e-20] |
| *Streptococcus* | *S. pyogenes* |  | M1_476 | AOI_1;orf016 | Unmodified | Putative_bacteriocin[4e-27] |
| *Streptococcus* | *S. pyogenes* |  | M1_476 | AOI_1;orf017 | Unmodified | Bacteriocin_likepeptide_associated[4e-41] |
| *Streptococcus* | *S. pyogenes* |  | M1_476 | AOI_1;orf021 | Unmodified | Mutacin_IV[4e-11] |
| *Streptococcus* | *S. pneumoniae* |  | SPN032672 | AOI_1;orf013 | Unmodified | Blpu[4e-31] |
| *Streptococcus* | *S. pneumoniae* |  | SPN032672 | AOI_3;orf001 | Unmodified | Blpi[8e-42] |
| *Streptococcus* | *S. pneumoniae* |  | SPN032672 | AOI_3;orf002 | Unmodified | Blpj[1e-54] |
| *Streptococcus* | *S. pneumoniae* |  | SPN032672 | AOI_3;orf003 | Unmodified | Blpk[2e-49] |
| *Streptococcus* | *S. pneumoniae* |  | SPN032672 | AOI_3;orf010 | Unmodified | Blpm[2e-57] |
| *Streptococcus* | *S. pneumoniae* |  | SPN032672 | AOI_3;orf011 | Unmodified | Blpn[7e-34] |
| *Streptococcus* | *S. pneumoniae* |  | SPN032672 | AOI_3;orf013 | Unmodified | Blpo[2e-32] |
| *Streptococcus* | *S. pneumoniae* |  | SPN032672 | AOI_4;orf010 | Unmodified | Lactococcin_972_(Lcn972)[5e-08 |
| *Streptococcus* | *S. pneumoniae* |  | SPN033038 | AOI_4;orf010 | Unmodified | Lactococcin_972_(Lcn972)[5e-08] |
| *Streptococcus* | *S. pneumoniae* |  | SPN033038 | AOI_3;orf009 | Unmodified | Blpi[8e-42] |
| *Streptococcus* | *S. pneumoniae* |  | SPN033038 | AOI_3;orf010 | Unmodified | Blpj[5e-58] |
| *Streptococcus* | *S. pneumoniae* |  | SPN033038 | AOI_3;orf011 | Unmodified | Blpk[2e-49] |
| *Streptococcus* | *S. pneumoniae* |  | SPN033038 | AOI_3;orf018 | Unmodified | Blpm[2e-57] |
| *Streptococcus* | *S. pneumoniae* |  | SPN033038 | AOI_3;orf019 | Unmodified | Blpn[7e-34] |
| *Streptococcus* | *S. pneumoniae* |  | SPN033038 | AOI_1;orf014 | Unmodified | Blpu[2e-26] |
| *Streptococcus* | *S. pneumoniae* |  | SPN994039 | AOI_1;orf013 | Unmodified | Blpo[4e-31] |
| *Streptococcus* | *S. pneumoniae* |  | SPN994039 | AOI_1;orf014 | Unmodified | Blpi[4e-41] |
| *Streptococcus* | *S. pneumoniae* |  | SPN994039 | AOI_2;orf010 | Unmodified | Lactococcin_972_(Lcn972)[6e-09 |
| *Streptococcus* | *S. pneumoniae* |  | SPN994039 | AOI_3;orf008 | Unmodified | Lactococcin_972_(Lcn972)[9e-14] |
| *Streptococcus* | *S. pneumoniae* |  | SPN034156 | AOI_1;orf012 | Unmodified | Lactococcin_972_(Lcn972)[6e-09] |
| *Streptococcus* | *S. pneumoniae* |  | SPN994038 | AOI_1;orf013 | Unmodified | Blpi[4e-41] |
| *Streptococcus* | *S. pneumoniae* |  | SPN994038 | AOI_1;orf014 | Unmodified | Blpo[4e-31] |
| *Streptococcus* | *S. pneumoniae* |  | SPN994038 | AOI_2;orf010 | Unmodified | Lactococcin_972_(Lcn972)[6e-09] |
| *Streptococcus* | *S. pneumoniae* |  | SPN994038 | AOI_3;orf008 | Unmodified | Lactococcin_972_(Lcn972)[9e-14] |
| *Streptococcus* | *S. pneumoniae* |  | SPN034183 | AOI_1;orf013 | Unmodified | Blpi[4e-41] |
| *Streptococcus* | *S. pneumoniae* |  | SPN034183 | AOI_1;orf014 | Unmodified | Blpo[4e-31] |
| *Streptococcus* | *S. pneumoniae* |  | SPN034183 | AOI_2;orf010 | Unmodified | Lactococcin_972_(Lcn972)[6e-09] |
| *Streptococcus* | *S. pneumoniae* |  | SPN034183 | AOI_3;orf008 | Unmodified | Lactococcin_972_(Lcn972)[9e-14] |
| *Streptococcus* | *S. agalactiae* |  | 2_22 | AOI_1;orf006 | Unmodified | Zoocin_A[3e-117] |
| *Streptococcus* | *S. iniae* |  | SF1 | AOI_1;orf008 | Unmodified | Lactococcin_972_(Lcn972)[2e-17] |
| *Streptococcus* | *S. iniae* |  | SF1 | AOI_2;orf007 | Unmodified | Thermophilin_13_chainb_(thmb)[2e-14 |
| *Streptococcus* | *S. iniae* |  | SF1 | AOI_3;smallORF_2 | LAPs |  |
| *Streptococcus* | *S. agalactiae* |  | 09mas018883 | AOI_1;orf007 | Bacteriocin >10kd | Zoocin_A[3e-117] |
| *Streptococcus* | *S. agalactiae* |  | ILRI005 | AOI_1;orf006 | Bacteriocin >10kd | Zoocin_A[3e-117] |
| *Streptococcus* | *S. agalactiae* |  | ILRI112 | AOI_1;orf007 | Bacteriocin >10kd | Zoocin_A[3e-117] |
| *Streptococcus* | *S. pyogenes* |  | HSC5 | AOI_1;orf014 | Unmodified | Blpu[4e-18] |
| *Streptococcus* | *S. pyogenes* |  | HSC5 | AOI_1;orf020 | Unmodified | Blpo[7e-07] |
| *Streptococcus* | *S. pyogenes* |  | HSC5 | AOI_2;orf009 | Unmodified | Putative_bacteriocin[1e-50] |
| *Streptococcus* | *S. pyogenes* |  | HSC5 | AOI_2;orf010 | Unmodified | Bacteriocin_likepeptide_associated[8e-44] |
| *Streptococcus* | *S. pyogenes* |  | HSC5 | AOI_2;orf011 | Unmodified | Mutacin_IV[3e-07] |
| *Streptococcus* | *S. pyogenes* |  | HSC5 | AOI_3;smallORF_3 | LAPs |  |
| *Streptococcus* | *S. lutetiensis* |  | 33 | AOI_2;orf011 | Bacteriocin >10kd | Zoocin_A[3e-24] |
| *Streptococcus* | *S. constellatus* | *pharyngis* | C232 | AOI_2;orf009 | Unmodified | Blpu[5e-08] |
| *Streptococcus* | *S. constellatus* | *pharyngis* | C232 | AOI_2;orf013 | Unmodified | Bovicin_255_variant[1e-25 |
| *Streptococcus* | *S. constellatus* | *pharyngis* | C232 | AOI_1;orf007 | LAPs |  |
| *Streptococcus* | *S. intermedius* |  | C270 | AOI_2;orf006 | Unmodified | Blpu[5e-24] |
| *Streptococcus* | *S. intermedius* |  | C270 | AOI_2;orf010 | Unmodified | Blpo[2e-07] |
| *Streptococcus* | *S. intermedius* |  | C270 | AOI_2;orf012 | Unmodified | Hiracin_JM79[1e-11] |
| *Streptococcus* | *S. intermedius* |  | C270 | AOI_2;orf014 | Unmodified | Mutacin_IV[3e-16] |
| *Streptococcus* | *S. intermedius* |  | C270 | AOI_2;orf015 | Unmodified | Blpu[2e-08] |
| *Streptococcus* | *S. constellatus* | *pharyngis* | C1050 | AOI_1;smallORF_8 | LAPs |  |
| *Streptococcus* | *S. constellatus* | *pharyngis* | C1050 | AOI_2;orf011 | Unmodified | Blpu[5e-08] |
| *Streptococcus* | *S. constellatus* | *pharyngis* | C1050 | AOI_2;orf015 | Unmodified | Bovicin_255_variant[9e-26] |
| *Streptococcus* | *S. anginosus* |  | C238 | AOI_1;smallORF_8 | LAPs |  |
| *Streptococcus* | *S. anginosus* |  | C238 | AOI_2;orf005 | Unmodified | Blpu[9e-09] |
| *Streptococcus* | *S. anginosus* |  | C238 | AOI_2;orf009 | Unmodified | Mutacin_IV[3e-16] |
| *Streptococcus* | *S. anginosus* |  | C238 | AOI_2;orf012 | Unmodified | Bovicin_255_variant[9e-26] |
| *Streptococcus* | *S. anginosus* |  | C238 | AOI_2;orf016 | Unmodified | Blpu[4e-27] |
| *Streptococcus* | *S. anginosus* |  | C1051 | AOI_1;orf009 | Unmodified | Blpu[2e-08] |
| *Streptococcus* | *S. anginosus* |  | C1051 | AOI_1;orf010 | Unmodified | Bovicin_255_variant[1e-25] |
| *Streptococcus* | *S. anginosus* |  | C1051 | AOI_1;orf014 | Unmodified | Blpu[1e-24] |
| *Streptococcus* | *S. constellatus* | *pharyngis* | C818 | AOI_1;orf008 | LAPs |  |
| *Streptococcus* | *S. constellatus* | *pharyngis* | C818 | AOI_2;orf009 | Unmodified | Blpu[5e-08] |
| *Streptococcus* | *S. constellatus* | *pharyngis* | C818 | AOI_2;orf013 | Unmodified | Bovicin_255_variant[1e-25] |
| *Streptococcus* | *S. intermedious* |  | B196 | AOI_1;smallORF_7 | Lanthipeptide_class_I |  |
| *Streptococcus* | *S. intermedious* |  | B196 | AOI_2;orf003 | Unmodified | Blpu[1e-25] |
| *Streptococcus* | *S. intermedious* |  | B196 | AOI_2;orf011 | Unmodified | Thermophilin_A[1e-07] |
| *Streptococcus* | *S. intermedious* |  | B196 | AOI_2;orf013 | Unmodified | Thermophilin_13_chainb_(thmb)[1e-14] |
| *Streptococcus* | *S. intermedious* |  | B196 | AOI_2;orf014 | Unmodified | Thermophilin_A[1e-31] |
| *Streptococcus* | *S. intermedious* |  | B196 | AOI_3;orf014 | Sactipeptides | Subtilosin_A[8e-16] |
| *Streptococcus* | *S. intermedious* |  | B196 | AOI_3;smallORF_8 | Sactipeptides | Subtilosin_A[7e-16] |
| *Streptococcus* | *S. dysgalactiae* | *equisimilis* | 167_DNA | AOI_1;smallORF_2 | Lanthipeptide_class_I |  |
| *Streptococcus* | *S. dysgalactiae* | *equisimilis* | 167_DNA | AOI_2;orf010 | Unmodified | Blpu[1e-09] |
| *Streptococcus* | *S. dysgalactiae* | *equisimilis* | 167_DNA | AOI_2;orf015 | Unmodified | Blpm[3e-33] |
| *Streptococcus* | *S. dysgalactiae* | *equisimilis* | 167_DNA | AOI_3;smallORF_6 | LAPs |  |
| *Streptococcus* | *S. dysgalactiae* | *equisimilis* | 167_DNA | AOI_4;orf012 | Unmodified | Ubericin-A[4e-25] |
| *Streptococcus* | *S. dysgalactiae* | *equisimilis* | I_G2 | AOI_1;orf009 | Sactipeptides | Subtilosin_A[2e-17] |
| *Streptococcus* | *S. pneumoniae* |  | A026 | AOI_1;orf010 | Unmodified | Blpu[4e-46] |
| *Streptococcus* | *S. pneumoniae* |  | A026 | AOI_2;orf010 | Unmodified | Lactococcin_972_(Lcn972)[1e-08] |
| *Streptococcus* | *S. pneumoniae* |  | A026 | AOI_3;orf012 | Unmodified | Blpo[2e-12] |
| *Streptococcus* | *S. pneumoniae* |  | A026 | AOI_3;orf013 | Unmodified | Acidocin_LF221B(gassericink7b)[3e-11] |
| *Streptococcus* | *S. pneumoniae* |  | A026 | AOI_3;orf024 | Unmodified | Blpm[6e-24] |
| *Streptococcus* | *S. pneumoniae* |  | A026 | AOI_4;orf011 | Unmodified | Lactococcin_972_(Lcn972)[3e-09] |
